# Evolving polarisation of infiltrating and alveolar macrophages in the lung during metastatic progression of melanoma suggests CCR1 as a therapeutic target

**DOI:** 10.1038/s41388-022-02488-3

**Published:** 2022-10-14

**Authors:** Thomas T. Tapmeier, Jake H. Howell, Lei Zhao, Bartlomiej W. Papiez, Julia A. Schnabel, Ruth J. Muschel, Annamaria Gal

**Affiliations:** 1 CRUK/MRC Oxford Institute for Radiation Oncology, Department of Oncology, University of Oxford, Oxford, OX3 7DQ UK; 2 Department of Obstetrics and Gynaecology, Monash University, Clayton, VIC 3168 Australia; 3 The Ritchie Centre, Hudson Institute of Medical Research, Clayton, VIC 3168 Australia; 4 School of Applied Sciences, University of Brighton, Brighton, BN2 4GJ UK; 5 Shandong Cancer Hospital and Institute, Shandong Cancer Hospital Affiliated to Shandong First Medical University, Jinan, 250117 China; 6 Li Ka Shing Centre for Health Information and Discovery, Big Data Institute, Oxford, OX3 7LF UK; 7 School of Biomedical Imaging and Imaging Sciences, King’s College London, London, SE1 7EU UK; 8 Helmholtz Center Munich – German Center for Environmental Health, 85764 Neuherberg, Germany; 9 Faculty of Informatics and Institute for Advanced Study, Technical University of Munich, 85748 Garching, Germany

**Keywords:** Immunosurveillance, Innate immune cells, Cancer models

## Abstract

Metastatic tumour progression is facilitated by tumour associated macrophages (TAMs) that enforce pro-tumour mechanisms and suppress immunity. In pulmonary metastases, it is unclear whether TAMs comprise tissue resident or infiltrating, recruited macrophages; and the different expression patterns of these TAMs are not well established. Using the mouse melanoma B16F10 model of experimental pulmonary metastasis, we show that infiltrating macrophages (IM) change their gene expression from an early pro-inflammatory to a later tumour promoting profile as the lesions grow. In contrast, resident alveolar macrophages (AM) maintain expression of crucial pro-inflammatory/anti-tumour genes with time. During metastatic growth, the pool of macrophages, which initially contains mainly alveolar macrophages, increasingly consists of infiltrating macrophages potentially facilitating metastasis progression. Blocking chemokine receptor mediated macrophage infiltration in the lung revealed a prominent role for CCR2 in Ly6C^+^ pro-inflammatory monocyte/macrophage recruitment during metastasis progression, while inhibition of CCR2 signalling led to increased metastatic colony burden. CCR1 blockade, in contrast, suppressed late phase pro-tumour MR^+^Ly6C^-^ monocyte/macrophage infiltration accompanied by expansion of the alveolar macrophage compartment and accumulation of NK cells, leading to reduced metastatic burden. These data indicate that IM has greater plasticity and higher phenotypic responsiveness to tumour challenge than AM. A considerable difference is also confirmed between CCR1 and CCR2 with regard to the recruited IM subsets, with CCR1 presenting a potential therapeutic target in pulmonary metastasis from melanoma.

## Introduction

The accumulation of macrophages in tumours predicts poor patient prognosis [1–3]. Tumor-associated macrophages (TAMs) promote tumour growth through various means, including secretion of proangiogenic factors, enhancement of tumour survival and tissue invasion, suppression of anti-tumour immunity, and resistance to chemotherapy [4–6]. The phenotype of TAMs has been described as consistent with the alternatively activated macrophage phenotype (M2) based on the M1 (classical activation) – M2 paradigm of macrophage polarisation. However, growing evidence from in vivo and clinical studies indicates that the plasticity of TAMs occurs on a much broader scale, defying the description as solely M2 [7–10].

Most cancer patients succumb to tumour metastasis rather than their primary tumour [11]. Mononuclear cells of the myeloid lineage, monocytes and macrophages, play an indispensable role in the development and progression of metastatic cancer [12–16]. A plethora of cytokines and chemokines have been implicated in the recruitment of TAMs [17–20]. The CC chemokines CCL2, CCL4 and CCL5 are potent monocyte chemoattractants functioning by activating their cognate receptors, mainly CCR2, CCR5 and CCR1/CCR5, respectively. Blocking receptors implicated in macrophage recruitment has been proven to be a viable option to suppress TAM turnover and accumulation in primary and secondary tumours in mice [7, 21–23].

The interaction of CC chemokines and their cognate receptors expressed on macrophages has been demonstrated to promote metastasis. In a breast cancer model, CCL2 recruited CCR2-expressing inflammatory monocytes to the lung to facilitate pulmonary metastasis [24], while we have shown a similar role for CCL2 in liver metastasis of colorectal cancer [25, 26]. Consequently, the chemokine-chemokine receptor (CCL/CCR) axis within myeloid cells of the tumour microenvironment has come into focus as a promising target for cancer therapy, with clinical trials underway [27, 28].

Nonetheless, the role of the CCL/CCR signalling axis in metastasis is complicated by widespread redundancy between receptors and ligands [29, 30]. CCL2/CCR2 signalling has been shown to promote the activation of the CCL3 (MIP-1α)/CCR1 signalling cascade in breast cancer metastasis to the lung [19], leading to the retention of metastasis associated macrophages and colony growth. Interrupting the inhibition of CCL2/CCR2-mediated monocyte recruitment has been shown to lead to a deleterious metastatic overshoot [31].

In the lung, resident alveolar macrophages derive from erythro-myeloid progenitors in the yolk sac and are capable of self-renewal. Macrophages are also recruited from circulating monocytes, likely replenishing the interstitial macrophage population [32–34]. Conflicting reports assign alveolar macrophages pro-inflammatory or anti-inflammatory phenotypes not entirely compatible with the M1 or M2 classifications [35]. Although alveolar macrophages have been described as facilitating pulmonary metastasis [36, 37], their response to tumour challenge and their phenotypical and functional plasticity during metastasis are not well understood.

To characterise macrophage plasticity and their expression of targetable chemokine receptors, we analysed F4/80^+^CD11b^+^CD11c^-^ infiltrating macrophages (IM) and F4/80^+^CD11b^-^CD11c^+^ resident alveolar macrophages (AM) in a mouse model of melanoma metastasis to the lung [38–40]. We compared polarity and immune response gene signatures as well as CC chemokine receptor expression of IM and AM at early and late phases of metastatic growth. We then determined the effect of CC chemokine receptor antagonists on the development and progression of lung metastasis and the pulmonary macrophage compartments over time.

We hypothesise that CCR inhibition will lead to significant alterations in pro-tumour and/or anti-tumour macrophage (IM) recruitment depending on the CCRs expressed and employed by the different macrophage populations, and that this would result in enhanced or reduced metastatic colony growth in the lungs. We also hypothesise that changes in IM recruitment in response to CCRi would bring along concomitant alterations in the AM population.

## Results

### Reference gene signatures of in vitro polarised macrophages and TAMs from subcutaneous melanoma allografts

We generated reference gene signatures from in vitro polarised bone marrow derived macrophages (BMDM) and TAMs isolated from subcutaneous (s.c.) B16F10 melanoma tumours from the same C57BL/6 mouse strain used in our subsequent metastasis studies. Because surface markers for the diverse macrophage phenotypes remain ambiguous [41], we analysed macrophage gene expression instead, using a custom panel of 73 putative marker genes by RT-qPCR (Table S1).

**Fig. 1:**
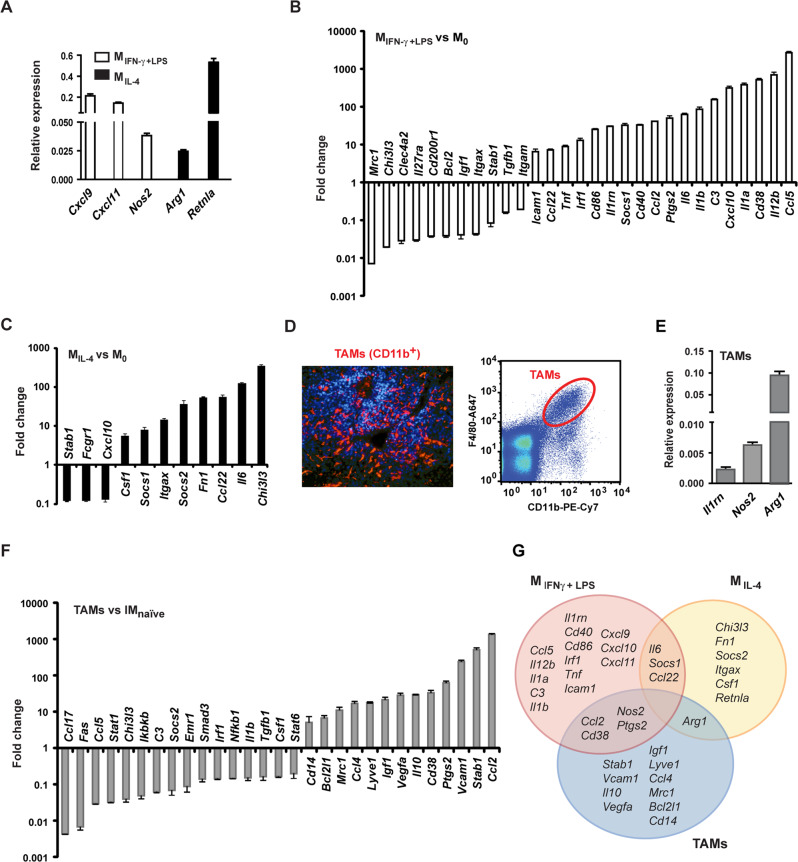
Polarity and immune response gene signatures of in vitro polarised macrophages and TAMs from s.c. melanoma. **A**–**C** Macrophages differentiated from the bone marrow of C57BL/6 mice were polarised in vitro toward M1 with 20 ng/ml IFN-γ plus 0.1 μg/ml LPS (M_IFN-γ+LPS_) and toward M2 with 20 ng/ml IL-4 (M_IL-4_) for 36 h, or left unpolarised. Total RNA was isolated and subjected to a customised RT-qPCR array of mouse immune response and macrophage polarity genes. Newly induced genes are displayed as average relative expressions + standard deviation (SD), *n* = 3 (**A**). Relative expression of regulated genes in M_IFN-γ+LPS_ (**B**) and in M_IL-4_ (**C**) is displayed as fold changes over the relative gene expression of unpolarised macrophages. Means of changes of 5-fold or greater + SD are shown, *n* = 3. **D** A representative image of CD11b immunostaining (red) of s.c. B16F10 melanoma from C57BL/6 mouse (nuclei are stained blue with DAPI), and a representative dot plot of fluorescence activated cell sorting of F4/80^+^CD11b^+^ TAMs from the tumour. **E**, **F** Total RNA was isolated from pooled TAMs of s.c. tumours (*n* = 3) and subjected to a customised RT-qPCR array of mouse immune response and macrophage polarity genes. Newly induced genes are displayed as average relative expressions + SD, *n* = 3 (**E**). Relative expression of regulated genes is shown as fold changes over the relative gene expression of *naïve* infiltrating (interstitial) lung macrophages. Means of changes of 5-fold or greater + SD are presented, *n* = 3 (**F**). **G** Venn diagram showing the overlap between upregulated genes of M_IFN-γ+LPS_, M_IL-4_ and TAMs.

BMDM from C57BL/6 mice were polarised in vitro toward M1 with IFN-γ + LPS or toward M2 with IL-4. The relative gene expression of polarised macrophages was compared with that of naïve BMDM. IFN-γ + LPS stimulation induced the expression of prominent pro-inflammatory chemokine genes, *Cxcl9* and *Cxcl11* (Fig. 1A) and upregulated *Ccl5*, *Cxcl10, Ccl2* and the pro-inflammatory cytokine genes, *Il12b, Il1a* and *Il1b*. M1 markers, such as *Cd38, Ptgs2* and *Cd40* were also upregulated (Fig. 1B) [42–44]. M2 markers, notably the mannose receptor gene (*Mrc1*) and *Chi3I3*, were downregulated (Fig. 1B).

IL-4 substantially induced the distinctive mouse M2 marker *Retnla* (*Fizz-1*) (Fig. 1A) and upregulated, among others, *Chi3I3*, *Fn1* and *Itgax* with concomitant downregulation of *Fcgr1* and *Cxcl10* (Fig. 1C) displaying the expected anti-inflammatory pattern. *Il-6* was upregulated in both conditions (Fig. 1B, C). These patterns are consistent with M1 and M2 polarisation as previously described [44]. The genes differentially regulated in M1 vs. M2 were *Cxcl10*, *Chi3l3* and *Itgax* (Fig. S1A).

Relative gene expression in F4/80^+^CD11b^+^ TAMs sorted from s.c. B16F10 tumours growing in C57BL/6 mice (Fig. 1D) was compared with that of F4/80^+^CD11b^+^ interstitial macrophages sorted from the lungs of naïve mice. TAMs showed increased expression of the M2-associated gene *Arg1* (Fig. 1E). *Ccl2*, an M1 marker in our model, was upregulated to the greatest extent emphasising the function of CCL2 in persistent recruitment of TAMs (Fig. 1F). Genes associated with cell adhesion and migration, including *Stab1*, *Vcam1* and *Lyve1*, as well as the angiogenic factor *Vegfa* and the proliferation/survival factor *Igf1*, and also *Mrc1* were among the upregulated genes (Fig. 1F). The most downregulated was the T-cell attracting pro-inflammatory chemokine gene, *Ccl17* [45] (Fig. 1F). These results confirmed a mixed phenotype of TAMs, consisting of both M1 and M2 subsets, and upregulating genes with known tumour promoting functions (Figs. 1G, S1B).

Next, we used the gene expression profiles of M_IFN-γ+LPS_, M_IL-4_ and TAMs as references for macrophage phenotyping in the experimental lung metastasis model.

### Polarisation of IM and AM in the progression of melanoma lung metastasis

**Fig. 2:**
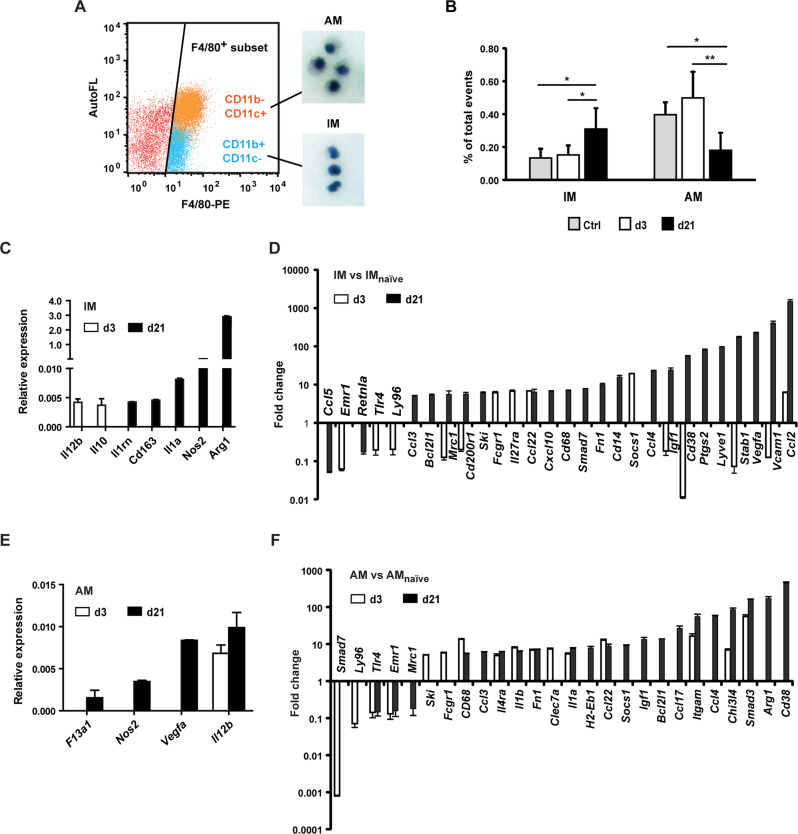
Polarity and immune response gene signatures of IM and AM in the early and late stage of pulmonary metastasis. **A** Macrophages were sorted from the lungs of C57BL/6 mice at d3 or d21 following i.v. B16F10 cell challenge. CD11b^+^CD11c^-^ and CD11b^-^CD11c^+^ cell subsets back-gated on the F4/80^+^ cell population were identified as infiltrating (IM) and alveolar macrophages (AM), respectively, following cytospin preparations. **B** Quantification of IM and AM sorted from cell suspensions of unchallenged (control) lungs or from lungs at d3 or d21 after i.v. injection of B16F10 cells. The macrophage numbers are shown relative to the total cell counts of individual lungs (% of total events). Bars represent the means + SD, *n* = 4–6 per group. To assess the differences among the means, one-way ANOVA and Tukey’s post-hoc tests were performed, *p < 0.05, **p < 0.01. **C–F** Total RNA was isolated from pooled IM or AM of naïve or metastasis-bearing lungs (*n* = 5 per group) and subjected to a customised RT-qPCR array of mouse immune response and macrophage polarity genes. Expression of newly induced genes is displayed as average relative expression + SD, *n* = 3 (**C**, **E**). Relative expression of regulated genes in IM (**D**) and AM (**F**) at the early or late stage of lung metastasis is displayed as fold changes over the relative gene expression of IM and AM from unchallenged lungs. Means of changes of 5-fold or greater + SD are shown, *n* = 3.

After intravenous (i.v.) injection of B16F10 melanoma cells in C57BL/6 mice, lungs were harvested at d3 when phagocytosis of tumour cells was evident (early stage), or at d21 at which time macroscopic colonies were present (late stage) (Fig. S2A). F4/80^+^CD11b^+^CD11c^-^ and F4/80^+^CD11b^-^CD11c^+^ cells were sorted from cell suspensions of the murine lungs (Fig. S2B), with both populations showing the characteristic morphologies of IM and AM, respectively (Fig. 2A). The AM population was also mannose receptor (MR) positive (Fig. S5). The proportion of IM at the early stage was similar to that of the naïve control, but significantly increased by the late stage. On the other hand, the frequency of AM significantly declined by the late stage compared with the control and d3 (Fig. 2B).

We then compared the gene expression of IM and AM in the early and late stages of pulmonary metastasis with gene expression of IM and AM from unchallenged lungs.

Early IM showed upregulation of *Socs1* and downregulation of *Cd38*, *Igf1* and *Mrc1* among other genes, consistent with polarisation toward an M1/pro-inflammatory phenotype (Fig. 2D). During the late phase of metastatic growth, *Arg1 was* substantially induced in IM (Fig. 2C). Upregulation of genes involved in macrophage recruitment (*Ccl2*), cell adhesion and migration (*Vcam1, Stab1, Lyve1*), angiogenesis (*Vegfa*) and cell proliferation/survival *(Igf1)* all suggested a phenotype similar to TAMs of s.c. melanoma (Fig. 2D). The genes differentially regulated between early and late stage IM were predominantly TAM markers (Fig. S1C).

In AM, we found a consistent upregulation of pro-inflammatory genes, such as *Il-12b, Il-1a* and *Il-1b*, and also anti-inflammatory genes, including *Smad3* of the TGF-β signalling pathway, and a considerable downregulation of the inhibitory Smad, *Smad7* from the early to the late stage (Fig. 2E, F). At the late stage, a mixed phenotype persisted. Some of the TAM markers were found upregulated, including *Vegfa*, *Cd38* and *Arg1* (Fig. 2E, F), while other genes including *Ccl17*, *Il-1b* and *Mrc1* were differentially regulated between TAMs and late stage AM (Fig. S1D).

**Fig. 3:**
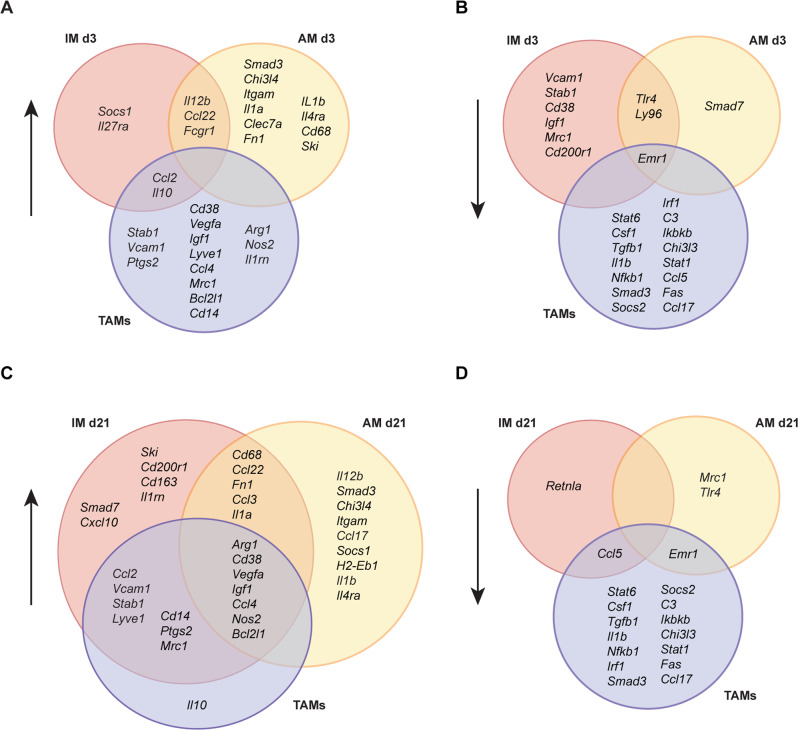
Comparison of gene expression of TAMs with IM and AM at the early or late stage of pulmonary metastasis. **A**, **B** Venn diagrams showing the overlap between upregulated (**A**) or downregulated (**B**) genes of TAMs from s.c. melanoma and IM and AM at the early stage of pulmonary metastasis. **C**, **D** Venn diagrams displaying the overlap between upregulated (**C**) or downregulated (**D**) genes of TAMs from s.c. melanoma and IM and AM at the late stage of pulmonary metastasis. Upwards arrows indicate gene upregulation and downwards arrows show gene downregulation.

In summary, the early gene expression of IM showed a pro-inflammatory pattern, while the early-stage AM displayed a mixed response, both with an insignificant overlap with the TAM profile (Fig. 3A, B). At the late stage, IM and AM shared twelve upregulated genes, seven of which are TAM markers (Fig. 3C). IM substantially mirrored the TAM signature at the late stage, whereas AM did so only partially (Fig. 3C, D).

### CC chemokine secretion in melanoma lung metastasis and cognate chemokine receptor expression of pulmonary macrophages

**Fig. 4:**
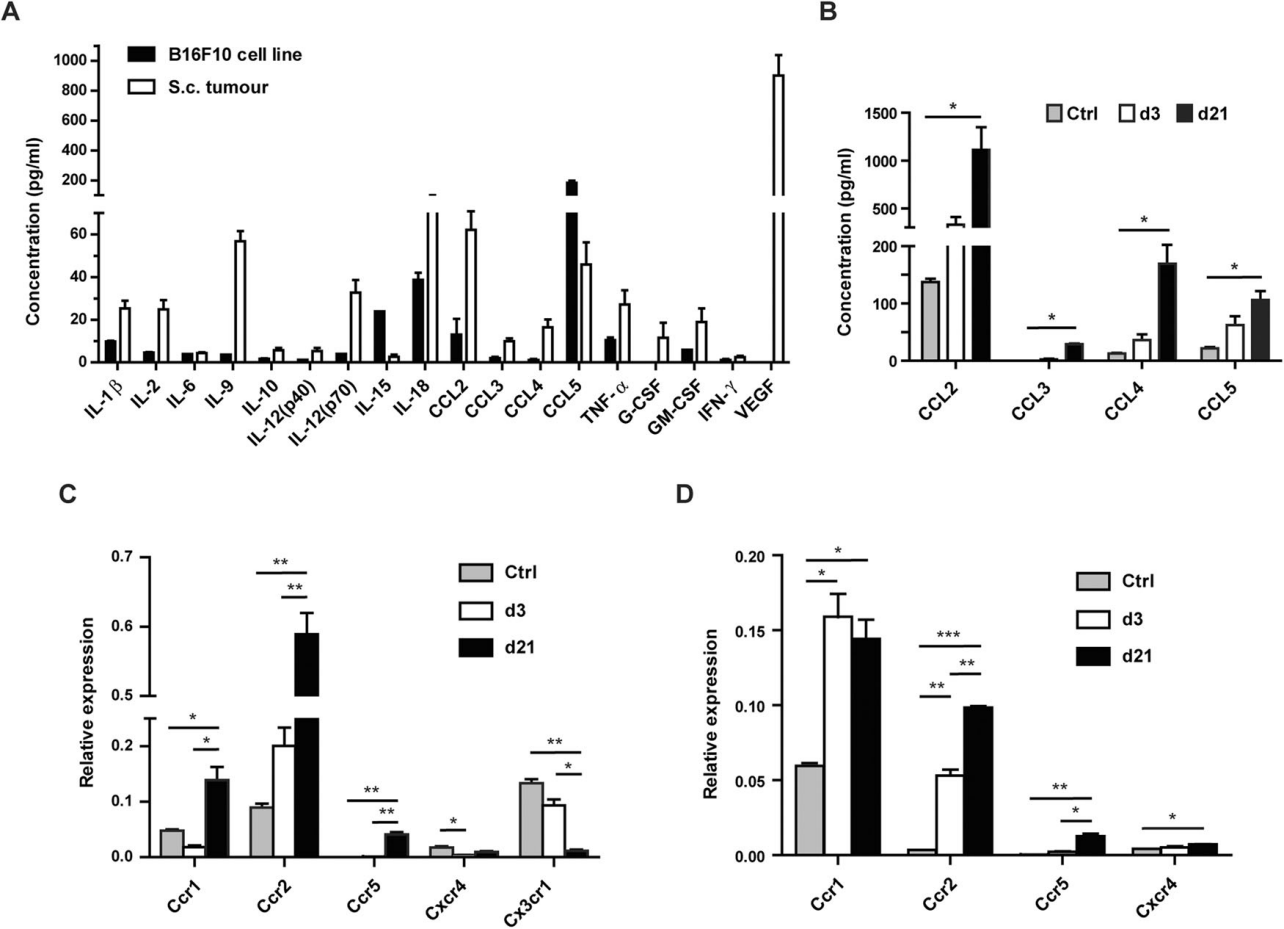
CC chemokine levels increase and the expression of their cognate receptors is upregulated in macrophages during pulmonary metastasis progression. **A** CM from B16F10 cell cultures or from single cell suspensions of ex vivo s.c. B16F10 tumours were harvested after 24 h incubation. Cytokine and chemokine concentrations were determined by Luminex assay and normalised to cell numbers. Bars represent the means + SD, *n* = 3 (cell culture) and *n* = 4 (tumour). **B** Blood was taken via cardiac puncture from unchallenged (control) mice (*n* = 3) and from lung metastasis-bearing mice at d3 or d21 (*n* = 5 per group). The sera were subjected to Luminex assay to determine CC chemokine concentrations. Bars show the means + SD, *n* = 3. To assess the differences of the means, Kruskal-Wallis and Bonferroni’s post-hoc tests were carried out, *p < 0.05. **C**, **D** Expression of chemokine receptors of pooled IM (**C**) or AM (**D**) isolated from metastasis-bearing lungs at d3 or d21, or from unchallenged (control) lungs (*n* = 5 per group), was determined in RT-qPCR assays. The means of relative gene expression + SD are shown, *n* = 3. Note the difference between the scales of the Y-axes in **C** and **D**.

To search for macrophage-recruiting chemokines in metastatic melanoma, we examined the secretion profiles of cultured B16F10 cell of cell cultures from melanoma tumours and in sera of mice with lung metastases. The B16F10 cells produced large amounts of CCL5/RANTES (Fig. 4A), whereas the ex vivo s.c. tumour also secreted CCL2/MCP-1 and, at much lower levels, CCL3/MIP-1α and CCL4/MIP-1β. However, the secretion profile was dominated by VEGF and also included inflammatory cytokines, among them IL-9 and IL-18 (Fig. 4A). CCL2 had the highest serum level in naïve mice, followed by CCL4 and CCL5, and their concentrations increased from the early to late stage of metastasis, just like in mice with the s.c. tumours (Figs. 4B, 3SC). A variety of cytokines were also detected at increasing levels in the sera of pulmonary metastasis-bearing mice, revealing a secretion pattern similar to that of s.c. melanoma-bearing mice (Figs. S3A, S3B). The VEGF serum level showed a considerable increase from the early to late stage of lung metastasis (Fig. S3A). Overall, CCL2 turned out to be the predominant macrophage recruiting CC chemokine observed during metastasis progression, followed by CCL4 and CCL5.

Next, we analysed the gene expression of CCR1, CCR2 and CCR5, the receptors activated by CCL5, CCL2 and CCL4/5, respectively, in IM and AM from metastases-bearing lungs. The expression levels of all three CC chemokine receptors increased, whereas the expression levels of other chemokine receptors like *Cxcr4* or *Cx3cr1* decreased in IM during metastasis progression (Fig. 4C).

In AM, *Ccr1* was the most prominent CC chemokine receptor gene expressed, with a similar expression level as in IM (Fig. 4D). By the late stage of metastasis, the expression levels of *Ccr1*, *Ccr2* and *Ccr5* had increased. In contrast, *Cx3cr1* was not expressed, and *Cxcr4* expression remained low (Fig. 4D).

### CCR2 inhibition enhances whereas CCR1 blockade reduces pulmonary metastatic burden

To block CCR1, CCR2 and CCR5, we used the selective chemokine receptor antagonists J-113863 [46] (CCR1i), RS-504393 [47] (CCR2i) and DAPTA [48] (CCR5i), respectively. Treatment was started one day before i.v. injection of B16F10 cells and repeated daily until termination of the experiment at d21 (Fig. S4A). We confirmed by flow cytometry that the CCR antagonists lowered the expression levels of the corresponding CCRs on F4/80^+^ pulmonary macrophages and/or reduced the quantity of macrophages expressing these CCRs, both at d3 and d21 (Fig. S4B).

**Fig. 5:**
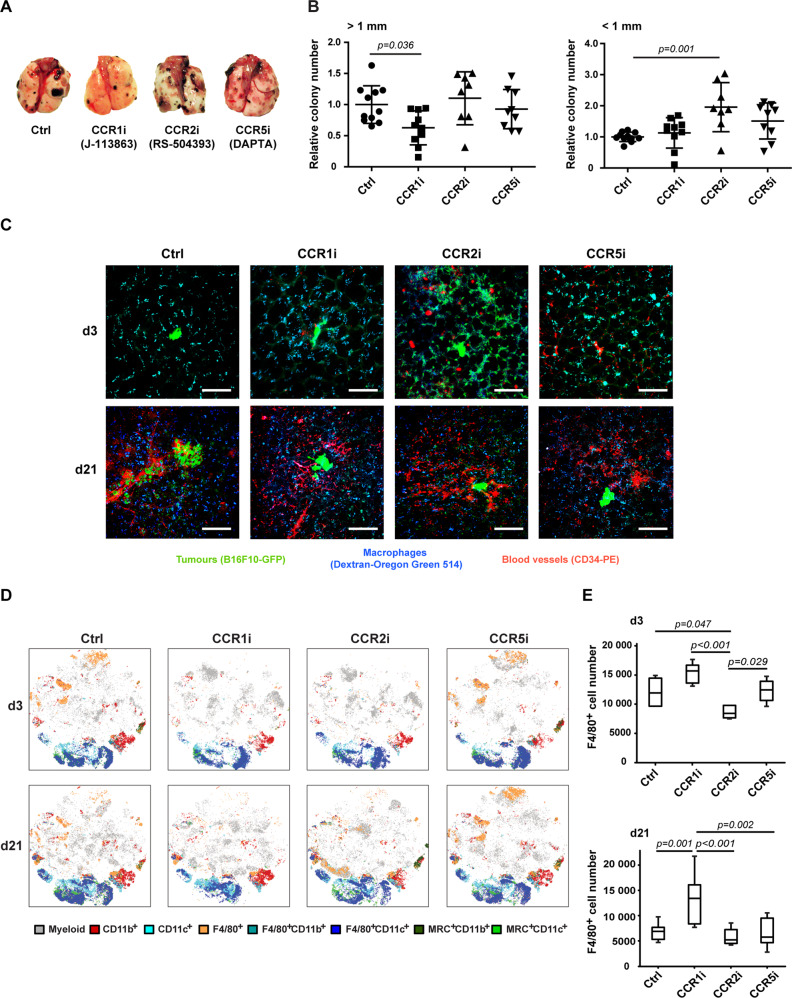
CCR1 blockade reduces metastatic burden in the lung whereas CCR2 inhibition increases it. **A** Representative images of lungs with surface colonies harvested at d21 after i.v. B16F10 cell injection with daily chemokine receptor inhibitor treatments targeting CCR1 (J-113863), CCR2 (RS-504393) or CCR5 (DAPTA). **B** Lung colonies with diameters > 1 mm or < 1 mm were quantified and expressed as colony numbers relative to the control. The bars represent the means ± SD *n* = 8–11 per group. For statistical analysis, one-way ANOVA with Dunnett’s post-hoc test (for >1 mm colonies) and Kruskal-Wallis test with Dunn’s multiple comparison test on selected pairs (for < 1 mm colonies) were performed. **C** C57BL/6 mice were injected into the tail vein with 2 × 10^5^ B16F10-GFP cells. Twenty-four hours before sacrifice at d3 or d21, mice were i.v. injected with Dextran-Oregon Green 514, and 1 h before sacrifice also injected with a PE-conjugated anti-CD34 antibody. The harvested whole lungs were imaged using confocal microscopy. Macrophages are blue (Oregon Green 514), tumour cells are green (GFP), and the vasculature is red (PE). Size bars indicate 100 μm. At d3, the first established small colonies are shown except for the CCR5i-treated lung where a colony is not visible. Smaller colonies at d21 can be seen in the CCR1i, CCR2i and CCR5i-treated lungs, whereas a larger one in the control. **D** Myeloid cell populations in the lungs of control or CCRi-treated mice were analysed by flow cytometry using a panel of myeloid cell markers. The analysis of samples, concatenated by treatment group using the visual stochastic neighbour embedding (viSNE) algorithm [76], revealed differences in the development of myeloid populations over the course of colony development, with CCR5 inhibition showing the least difference compared with controls. CCR1 and CCR2 inhibition both led to a markedly different composition of myeloid populations, especially in the F4/80^+^CD11c^+^ (AM) compartment (dark blue), *n* = 5–10 per group at day 3, *n* = 9–14 per group at day 21. **E** F4/80^+^ macrophages were quantified by flow cytometry in cell suspensions of control or CCRi-treated lungs (10^5^ total events) at d3 and d21. Means ± SD are shown, *n* = 5 per group at d3, and *n* = 8–11 per group at d21. To determine the differences among the means, one-way ANOVA and Tukey’s post-hoc tests were performed.

CCR1 blockade resulted in a decreased number of large (>1 mm) pulmonary colonies compared with the control. In contrast, blocking CCR2 did not significantly affect the number of large colonies, but led to an increase in the number of small (<1 mm) colonies compared with the control (Fig. 5A, B).

Via ex vivo imaging of the lungs, we observed an increase in the total macrophage numbers after CCR1i treatment both at d3 and d21 compared with the other experimental groups (Fig. 5C). CCR2 inhibition resulted in a larger number of tumour cells at d3 and more extensive vascularisation at d21 than observed in the other groups. Lungs treated with CCR5i did not show significant alterations compared with the control (Fig. 5C).

The comparison of global changes within the myeloid compartment over time with and without CCR inhibition showed little difference between populations of the control and CCR5i group, but marked differences in the AM population following CCR1i and CCR2i treatments (Fig. 5D). Quantification of F4/80^+^ pulmonary macrophages by flow cytometry revealed a reduced number of F4/80^+^ cells at d3 following CCR2 inhibition and a significant increase in the F4/80^+^ cell number at d21 in response to CCR1i compared with the other groups, confirming this impression (Figs. 5E, S5). The total CD11c^+^ pulmonary cell population showed a significant expansion both at d3 and d21 when CCR1 was inhibited (Fig. S6A), whereas the total CD11b^+^ pulmonary cell number didn’t change significantly in response to any of the CCR antagonists compared with the control (Fig. S6B).

### CCR2 inhibition suppresses Ly6C^+^ IM recruitment whereas CCR1 blockade reduces the Ly6C^-^ IM subset and allows AM accumulation

**Fig. 6:**
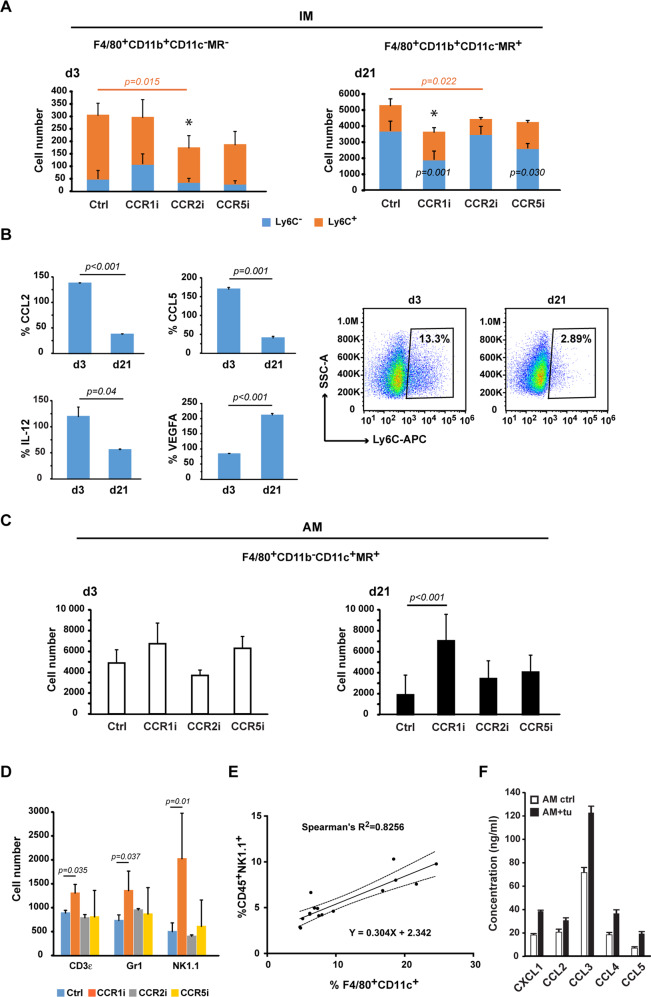
CCR1 inhibition reduces Ly6C^-^ IM recruitment leading to AM expansion and NK cell accumulation in the lung. **A** Flow cytometry analysis was performed to quantify the frequency of the MR^-^Ly6C^+^/Ly6C^-^ and MR^+^Ly6C^+^/Ly6C^-^ subsets of IM in 10^5^ total events in the lungs of the control or CCRi-treated mice. The bars represent the mean cell numbers + SD, *n* = 5 both at d3 and d21. The differences between the means were assessed by one-way ANOVA followed by Dunnett’s multiple comparison test between total IM of experimental groups and the respective controls, *p < 0.05. Significant differences between the Ly6C^+^ subsets are shown by orange lines with *p*-values, whereas significant differences between the Ly6C^-^ subsets of experimental groups and the respective control are indicated by *p*-values displayed across the blue bars. **B** The concentrations of the pro-inflammatory CCL2, CCL5 and IL-12 along with the pro-tumour VEGFA were determined by ELISA in the CM of AMJ2-C11 macrophages separated from their d3 or d21 co-culture with B16F10 cells and expressed as percentages of the monoculture concentrations. The bars represent the mean relative concentrations + SD, *n* = 3. The differences between the means were assessed by independent *t*-tests. Representative dot plots are showing the frequency of the Ly6C-expressing macrophages from the d3 and d21 co-cultures. **C** Flow cytometry analysis was performed to quantify the AM compartments following inhibition of CCRs. The frequency of AM in 10^5^ total events was expressed as mean AM numbers + SD, *n* = 5 per group at d3 and *n* = 7–11 per group at d21. One-way ANOVA was carried out followed by Dunnett’s multiple comparison test. **D** The frequency of T-cells, neutrophils and NK cells were quantified by flow cytometry in 10^5^ total events in lung cell suspensions at d21 using antibodies against CD3ε, Gr1 (Ly6G) and NK1.1, respectively. The bars indicate the mean cell numbers + SD, *n* = 5 per group. One-way ANOVA followed by Dunnett’s multiple comparison tests were performed. **E** The quantity of F4/80^+^CD11c^+^ AM and NK1.1^+^ NK cells expressed as % of viable cells in late-stage metastatic lungs correlate as shown by the combined d21 data, *n* = 16. Non-parametric Spearman’s correlation was carried out, *p* = 0.0002; linear regression with 95% CI. **F** Luminex assay was performed to determine the concentrations of chemokines in the conditioned medium of ex vivo AM cultures from unchallenged (AM ctrl) and B16F10-challenged (24 h) lungs (AM + tu), *n* = 3 each. The concentrations were normalised to cell numbers. The bars represent the average concentrations + SD.

Analysing the IM and AM compartments of F4/80^+^ pulmonary macrophages in response to the CCR antagonists in more detail, we observed that the IM population was significantly reduced by CCR2i at the early stage of lung metastasis, whereas at the late stage, the IM numbers were instead decreased by CCR1i compared with the respective controls (Fig. 6A). Combined with the gene expression profiling of IM (Fig. 2C–F), these results indicate that CCR2i inhibited pro-inflammatory whereas CCR1i reduced anti-inflammatory/pro-tumour IM recruitment to the lung. Aiming to identify IM subsets particularly affected by CCR2i and CCR1i, we focused on Ly6C-expressing macrophages. Ly6C^+^ IM have been implicated in pro-inflammatory responses [49]. Total Ly6C^+^ cell numbers were significantly reduced in response to each CCR antagonist at the early stage but decreased only by CCR2i at the late stage of lung metastasis compared with the control groups (Fig. S6C). The Ly6C^+^ fraction of IM significantly decreased while the Ly6C^−^ fraction significantly increased from d3 to d21 in all groups except from the CCR1i-treated one (Fig. S6D). Regarding the Ly6C^+^ IM subsets, we observed that CCR2i significantly lowered the numbers of MR^-^Ly6C^+^ IM at d3 (all IM were MR^-^ at this stage) as well as the MR^+^Ly6C^+^ IM at d21 compared with the control (Figs. 6A, S5). CCR1i and CCR5i did not reduce the numbers of Ly6C^+^ IM either at the early or the late stage (Fig. 6A). Regarding the Ly6C^-^ IM subsets, CCR1i and CCR5i caused a significant reduction in the MR^+^Ly6C^−^ IM numbers at d21 compared with the control (Fig. 6A).

To demonstrate the pro-inflammatory traits of TAMs with increased Ly6C-expression [49] as well as the tumour promoting characteristic of TAMs with reduced Ly6C-expression [50], we co-cultured the CD11b^+^Ly6C^+^ AMJ2-C11 syngeneic mouse macrophage cell line (Fig. S7A) with B16F10 cells for up to 21 days. We evaluated the Ly6C cell surface expression as well as chemokine and cytokine secretion of the AMJ2-C11 macrophages at d3 and d21. Secretion of the pro-inflammatory CCL2, CCL5 and IL-12 decreased with reduced expression of Ly6C. Conversely, secretion of the pro-tumour VEGFA increased with lower Ly6C expression, comparing the co-cultures at d3 and d21 (Fig. 6B).

The AM population showed a significant expansion at the late stage after CCR1 inhibition, but no other changes were observed in response to the CCR antagonists compared with the respective controls (Fig. 6C). To find out whether CCR1i increases the proliferation of AM, we treated the AMJ2-C11 monoculture and the d3/d21 co-cultures with CCR1i and quantified the Ki-67 expression of the macrophages. We saw a decrease in the Ki-67 positive fraction of the monoculture in response to CCR1i, and we also observed a decrease in the untreated co-cultures compared with the monoculture, but no substantial changes were found in the co-cultures following CCR1 blockade (Fig. S7B, C).

Assessing other immune cells in the lungs, we detected a significant increase in the accumulation of T-cells, neutrophils and NK cells after CCR1 inhibition at d21 compared with the respective controls (Figs. 6D, S5), indicating a shift towards a pro-inflammatory tumour microenvironment in this group. The size of the NK cell population correlated with the size of the AM population (Fig. 6E), suggesting an immunomodulatory function of AM in the lung microenvironment. Indeed, B16F10 challenge in the lung increased the secretion of chemokines by AM, including CCL3, CCL4 and CCL5 also recruit NK cells (Fig. 6F).

While focusing on the effects of CCRi on the sizes of pulmonary macrophage compartments, we also wanted to know how the CCR antagonists altered the serum concentrations of macrophage-recruiting chemokines. At the early stage of lung metastasis, CCR1i and CCR5i significantly reduced the concentrations of circulating CCL2 and CCL5. The level of CCL3 was also decreased by CCR5i (Fig. S8A). The serum levels of pro-inflammatory cytokines, including GM-CSF, TNF-α, IL-12 and IL-1α as well as the anti-inflammatory IL-10 and IL-13 were similarly reduced by CCR1i and CCR5i (Fig. S8A). By d21, the only significant effect of CCR inhibition was that of CCR1i reducing the concentration of circulating GM-CSF (Fig. S8B).

## Discussion

Here, we analysed differences in phenotypic plasticity and response to CCR inhibitors between the predominant infiltrating and resident pulmonary macrophage compartments in the course of metastasis progression in the lung. IM displayed a pro-inflammatory phenotype at the early stage which developed into an anti-inflammatory/pro-tumour phenotype at the later stage. On the other hand, TAMs from s.c. melanoma and AM from pulmonary metastasis showed a mixed pro-inflammatory and anti-inflammatory gene expression, likely due to macrophage subsets of different polarisation states. However, it also needs to be considered that a plethora of secreted molecules from cellular interplay within the tumour microenvironment impact macrophages simultaneously, irrespective of the M1/M2 paradigm, and that therefore macrophages can express markers associated with opposite ends of the polarisation spectrum.

Accordingly, Jones et al. showed that the same tumour-associated macrophages display both M1 and M2 markers [51]. A hybrid phenotype of steady-state human AM has also been reported, suggesting an ability of AM to quickly switch between M1 and M2 phenotype to allow appropriate functional adaptation in the tissue environment [52]. On the other hand, Chen et al. demonstrated that AM display a high resemblance to IL-10 activated macrophages [53]. Upregulation of *Ccl17* by AM represents an anti-tumour trait, consistent with reports showing that increased serum levels of CCL17 are associated with improved survival of advanced melanoma patients [54, 55].

Colonising melanoma cells and their microenvironment in the lung are sources of secreted factors enriching the serum and attracting monocytes to the metastatic site. Of the CC chemokines, CCL2, CCL4 and CCL5 have all been implicated in cancer development and metastasis formation [19, 24, 56–64]. We found CCL2 dominating the serum CC chemokine profile, and IM having increasing expression of *Ccr2* during metastatic growth, which indicates that CCL2/CCR2 is a major signalling axis for monocyte-derived IM recruitment in lung metastasis. Serum CCL4 and CCL5, activating their cognate receptors CCR5 and CCR1/CCR5, respectively, were also implicated in our study as important chemokine signalling pathways in IM recruitment. Therefore CCR1, CCR2 and CCR5 presented as candidate targets for preventing pro-tumourigenic IM accumulation in melanoma lung metastasis.

Because of its primary role in macrophage chemotaxis, the CCL2/CCR2 chemokine signalling pathway has been frequently studied in mouse models of cancer as well as in cancer patients, with translation into the clinic. CCR2 antagonists have been found to suppress hepatocellular carcinoma growth [65], reduce lung metastasis from LLC tumours [66] and liver metastases from colorectal [67] or breast cancer xenografts [68] in animal models. A phase II clinical trial (NCT01015560) of the anti-CCR2 antibody MLN1202 showed efficacy in 14% of patients with bone metastasis [69]. However, in metastatic castration-resistant prostate cancer, carlumab, a human monoclonal antibody against CCL2, did not produce a therapeutic effect (NCT00992186) [70].

In our study, the selective CCR2 antagonist RS-504393 increased the metastatic burden in the lungs. Administration of CCR2i suppressed Ly6C^+^ pro-inflammatory IM recruitment both at the early and the late stage of metastasis, resulting in a significantly higher number of small colonies. As CCR1i and CCR5i did not have a similar effect to CCR2i, these results confirm that CCR2 is crucially involved in the recruitment of the Ly6C^+^ IM subsets [49].

In previous studies, CCR1 blockade with the antagonist CCX721 reduced tumour burden and osteolysis in a mouse model of myeloma bone disease [71], while another CCR1 antagonist, BL5923, was able to suppress liver metastasis from colorectal cancer [72].

In our study, CCR1 blockade with the antagonist J-113863 led to a reduced metastatic tumour burden due to fewer large colonies. Although both CCR1i and CCR5i caused a significant decrease in pro-tumour MR^+^Ly6C^-^ IM numbers at the late stage compared with the control, in the CCR1i treated group this ensured an unchanged frequency of pro-tumour Ly6C^-^ cells within the total IM population from d3 to d21, while in the CCR5i treated group the Ly6C^-^ IM frequency significantly increased from d3 to d21. This might be one reason for the lack of suppression of lung colony burden by CCR5i. Moreover, the ratio of Ly6C^+^ IM to total IM significantly decreased from d3 to d21 in the CCR5i group, but not in the CCR1i group. At the late stage, suppression of the pro-tumour IM could be a reason for the expansion of the AM compartment by CCR1i due to enhanced AM survival. However, testing in vitro, we did not find compelling evidence for CCR1i increasing AM proliferation. The expansion of AM with its partial pro-inflammatory phenotype correlated with the accumulation of NK cells and was accompanied by increased recruitment of inflammatory cells, which explains the significantly lower metastatic burden in the CCR1i treated group.

Our findings of reduced serum levels of pro-inflammatory cytokines and chemokines in response to CCR1i and CCR5i suggest that the CCR1 and CCR5 blockade has predominantly anti-inflammatory effects at the early stage of lung metastasis. However, the reduced chemokine and cytokine serum concentrations do not seem to affect macrophage recruitment. On the other hand, the significant decrease in GM-CSF serum levels by CCR1i at the late stage of lung metastasis might have contributed to the decreased MR^+^Ly6C^-^ IM number and reduced metastatic burden in response to CCR1i. This needs further investigation.

**Fig. 7:**
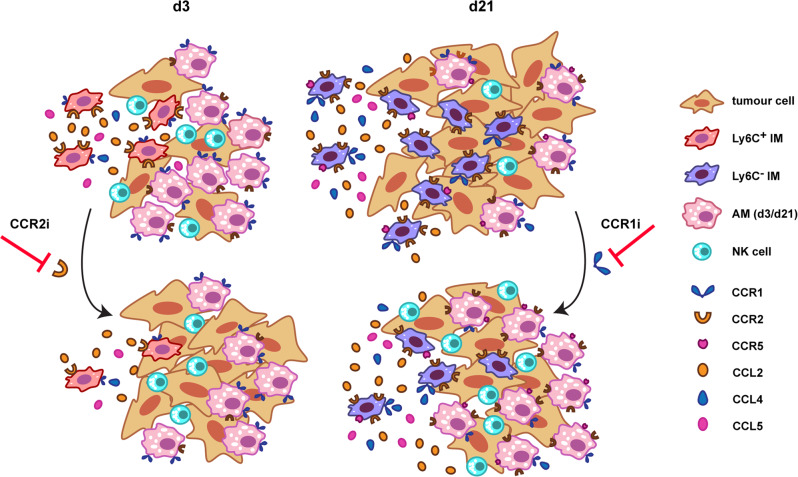
Summary of immune cell infiltration in the early and late stage of pulmonary metastasis progression in response to CCR2 and CCR1 inhibition. Blocking CCR2 inhibits the recruitment of Ly6C^+^ pro-inflammatory macrophages to the lung early after B16F10 tumour cell challenge, which results in an increase in pulmonary colony formation. On the other hand, CCR1 inhibition suppresses MR^+^Ly6C^-^ pro-tumour IM recruitment and augments the AM compartment at the later stage, and is accompanied by accumulation of NK cells, thereby reducing metastatic burden.

In summary, we propose that inhibition of CCR2 reduces the recruitment of early-stage MR^-^Ly6C^+^ pro-inflammatory macrophages, which leads to enhanced survival and progression of metastasising melanoma cells. CCR1 inhibition, on the other hand, suppresses the accumulation of MR^+^Ly6C^-^ pro-tumour IM at later stages, accompanied by the expansion of the AM compartment and infiltration of NK cells, resulting in reduced metastatic burden (Fig. 7). Of the CC chemokine receptors examined, we have found CCR2 involved in pro-inflammatory Ly6C^+^ IM recruitment and CCR1 in pro-tumourigenic Ly6C^-^ IM accumulation and, possibly, in the regulation of the AM compartment. The exact mechanisms of how CCR1 blockade leads to the expansion of the AM population warrant further investigations, while the analysis of the effects of chemokine receptor inhibitors on the gene expression of pulmonary macrophage populations is beyond the scope of this study and the subject of future research.

CCR1 inhibitors, therefore, may have therapeutic potential in reducing the progression of melanoma lung metastasis.

## Materials and Methods

### Cell culture

Parental and GFP-expressing B16F10 cells and the L929 mouse fibroblast cell line (ATCC, LGC Standards, Teddington, UK) were maintained in high glucose (4.5 g/l) DMEM supplemented with 10% FBS and 1% Penicillin-Streptomycin 10,000 U/ml (all from Fisher Scientific, Loughborough, UK) in a humidified incubator at 37 °C in 5% CO_2_ atmosphere. The conditioned medium of L929 cells (LCM) was harvested from 70 to 80% confluent cultures after 10–11days of incubation in 50 ml growth medium/ T175. The LCM was centrifuged at 1200 RPM for 5 min, and the supernatant sterile filtered through a 0.22 μm filter. LCM was added to bone marrow derived cells to potentiate macrophage differentiation.

Bone marrow-derived macrophage culture (BMDM) was prepared as described previously [73].

### In vitro macrophage polarisation

BMDM was polarised to M1 by incubating the cells with 20 ng/ml IFN-γ (Thermo Fisher Scientific, Stone, UK) + 0.1 μg/ml LPS from E. coli O55:B5 (Sigma-Aldrich, Gillingham, UK), or to M2 with 20 ng/ml IL-4 (Thermo Fisher Scientific) in Opti-MEM (Thermo Fisher Scientific). The polarisation was carried out for 36 h.

### Animal studies

Female 8–12 week old C57BL/6 mice were obtained from Charles River UK, Ltd. (Margate, UK), housed in IVC units, and given food and water *ad libitum*. Animals were treated in accordance with the Animals (Scientific Procedures) Act 1986 and the University of Oxford ethical guidelines.

Mice were s.c. injected with 5 × 10^4^ B16F10 cells in 50 μl sterile PBS under general anaesthesia with Isoflurane. Tumour growth was monitored and the tumour size measured with a caliper twice per week. The tumour volume was calculated according to the formula V = π/6 × a^2^ × b, where ‘a’ and ‘b’ represent the shorter and longer dimensions of the tumour, respectively. Mice were sacrificed by intraperitoneal (i.p.) administration of Pentobarbital, and the tumours were excised before reaching 12.5 mm geometric mean diameter.

For the experimental lung metastasis model, mice were i.v. injected with 2 × 10^5^ B16F10 cells in 100 μl sterile PBS. Mice were sacrificed by cervical dislocation 3 or 21 days later, and the lungs were harvested. For ex vivo imaging, B16F10-GFP cells were injected.

### Chemokine receptor inhibition

The chemokine receptor antagonists J-113863, RS-504393 and DAPTA (TOCRIS, Bio-Techne Ltd., Abingdon, UK) were used to inhibit CCR1, CCR2 and CCR5, respectively. J-113863 was administered 10 mg/kg/day i.p., RS-504393 was given 4 mg/kg/day orally and DAPTA 0.01 mg/kg/day s.c. Mice were administered the antagonists daily from 24 h before i.v. injection of tumour cells until sacrifice at day 3 or day 21.

### Lung ex vivo microscopy

To visualise colony growth in the lung, mice were i.v. injected with 2 × 10^5^ B16F10-GFP cells. Macrophages were labelled with Oregon Green 514 by i.v. injecting the mice with Dextran-Oregon Green 514 (Fisher Scientific) 24 h before sacrifice. To label the blood vessels, a PE-conjugated anti-mouse CD34 antibody (clone MEC14.7, Fisher Scientific) was i.v. injected 1 h before sacrifice. Ex vivo imaging of the lungs was done as described earlier [74].

### Preparation of single cell suspensions from s.c. tumours and lungs

Excised s.c. tumours were pushed through a cell strainer, mesh size 70 μm (Fisher Scientific), to prepare single cell suspensions. The cells were then resuspended in FACS washing buffer (2% FBS, 0.05% sodium-azide in PBS) in the presence of 1 U/μl Superase.In RNase inhibitor (Fisher Scientific) for cell sorting and subsequent RNA isolation.

Digestion of the harvested lungs was carried out as described earlier [75].

### Fluorescence activated cell sorting

Single cell suspensions from s.c. tumours and control or metastasis-bearing lungs were incubated with an anti-mouse CD16/32 Fc-blocking antibody (clone 93, BioLegend, London, UK), and 1 × 10^6^ cells per sample were stained in 100 μl volume with anti-mouse-F4/80-PE (clone BM8, Fisher Scientific), anti-mouse CD11b-PE-Cy7 (clone M1/70, Fisher Scientific) and anti-mouse CD11c-A647 (clone N418, BioLegend) antibodies. From the s.c. tumours, the F4/80^+^CD11b^+^ cell population, whereas from the lungs, the F4/80^+^CD11b^+^CD11c^-^ infiltrating macrophages and the F4/80^+^CD11b^-^CD11c^+^ alveolar macrophages were sorted using a MoFlo XDP cell sorter (Beckman Coulter, High Wycombe, UK). For improved gating boundaries, fluorescence-minus-one (FMO) controls were used.

For details of flow cytometry of cell markers, see the Supplemental Materials & Methods.

### Gene expression array/assays

RNA was isolated from BMDM using the RNeasy Mini kit. For RNA isolation from macrophages of s.c. tumours or metastasis-bearing lungs, the RNeasy Micro kit was used (both kits from Qiagen, Manchester, UK) according to the manufacturer’s instructions, including on-column DNA digestion. RNA integrity was assessed in the Agilent 2100 Bioanalyzer (Agilent Technologies, Stockport, UK). From the RNA of BMDM or pooled macrophages from s.c. tumours, 5 μg was reverse transcribed using the Superscript III First-Strand Synthesis System (Fisher Scientific). RNA of pooled infiltrating or alveolar macrophages from metastasis-bearing lungs was reverse transcribed and subjected to cDNA amplification using the QuantiTect Whole Transcriptome Kit (Qiagen) according to the manufacturer’s protocol. The cDNA was added at 25–50 ng/20 μl/well in 96-well plates of a customised mouse AB TaqMan PCR array (Thermo Fisher Scientific) according to the manufacturer’s instructions. For determining the expression levels of chemokine receptors, AB TaqMan PCR assays were prepared (Thermo Fisher Scientific) following the manufacturer’s protocol. The array and the assays were run on an AB 7500 Real-Time PCR System with SDS Software v1.2.3 (Thermo Fisher Scientific). The relative abundance of specific mRNA levels was calculated by normalising it to the housekeeping genes (HKGs) *18* *S, Gapdh, Hprt1* and *Gusb* using the 2^−ΔΔCt^ method. *Gapdh* turned out to be the most stable HKG in our experiments. Since the majority of genes selected for our PCR array were not associated with hypoxia, *Gapdh* was used as HKG.

### Luminex assay

The media of in vitro polarised BMDM and B16F10 cell cultures (60–80% confluent) were changed for Opti-MEM, and the conditioned media (CM) harvested 24 h later. CM from ex vivo cultured alveolar macrophages were harvested 24 h after plating the macrophages in complete DMEM.

Blood was taken via cardiac puncture from euthanized mice bearing s.c. melanoma or lung metastases, and the sera were separated after letting the blood clot at room temperature for 30 min.

The CM and sera were cleared by centrifugation for 10 min at 12,000 RPM. The clear supernatants were subjected to Luminex assay using a Bioplex Mouse Group I 23-plex panel combined with single-plex components of IL-15, IL-18 and VEGF (BioRad, Watford, UK) on the Luminex 200 System according to the manufacturer’s protocol. Standard curves were optimised and protein concentrations calculated using the Bio-Plex Manager software v6.0 (BioRad) or Prism 8 (GraphPad, San Diego, USA).

### Statistical analysis

For the analysis of normally distributed data, unpaired Student’s t-test or one-way ANOVA were carried out where suitable, followed by Tukey’s or Dunnett’s multiple comparison tests. For not normally distributed data, Kruskal-Wallis with Bonferroni’s correction or Dunn’s tests were performed using Minitab 19 (Minitab Ltd., Coventry, UK) or the Prism 8 statistical software. A null hypothesis was rejected at less than 5% probability (*p* < 0.05).

The description of the AMJ2-C11 cell culture, Bronchoalveolar lavage, ELISA, Immunohistochemistry and Cytospin preparation can be found in the Supplemental Materials & Methods.

## Supplementary information

Supplementary Materials
