# Supplementary material for: Evolving polarisation of infiltrating and alveolar macrophages in the lung during metastatic progression of melanoma suggests CCR1 as a therapeutic target
Source: Oncogene. 2022 Oct 14;41(46):5032–45. doi: 10.1038/s41388-022-02488-3 (PMC9652148; doi:10.1038/s41388-022-02488-3)
Supplement: Supplementary file 1 — Supplementary Materials [file 41388_2022_2488_MOESM1_ESM.docx]

**Supplementary Materials**

The Supplementary Materials contain the Supplemental Materials & Methods, Supplementary Table S1 and Supplementary Figures S1-8 with legends.

**Supplemental Materials & Methods**

**AMJ2-C11 cell culture**

The AMJ2-C11 mouse macrophage cell line (ATCC, Teddington, UK) was maintained as a suspension culture in DMEM, and supplemented with 5% FBS and 1% Penicillin/Streptomycin (P/S) 10 000 U/ml (all from Fisher Scientific, Loughborough, UK) in a humidified incubator at 37 ⁰C in 5% CO_2_ atmosphere. To establish a co-culture, 2x10^6^ AMJ2-C11 cells were added to adherent B16F10 cells at 50% confluency in T75 flasks (Fisher Scientific) containing DMEM supplemented with 10% FBS and 1% P/S. The macrophages were harvested from the co-culture 3 or 21 days later. The co-culture was split when the B16F10 cells reached 80% confluency. The mono- and co-cultures were treated with 45 nM J-113863 (CCR1 antagonist) for 48 h, or left untreated (control), before flow cytometry was carried out to quantify Ki-67 expression.

**Bronchoalveolar lavage**

C57BL/6 mice were i.v. injected with 2x10^5^ B16F10 cells or left unchallenged (control). Alveolar macrophages were harvested from the lungs via bronchoalveolar lavage as described elsewhere^1^. The alveolar macrophages were cultured in DMEM supplemented with 10% FBS for 24 h, followed by harvesting the conditioned media (CM) for subsequent Luminex assay.

**Enzyme-linked immunosorbent assay (ELISA)**

AMJ2-C11 macrophages were harvested from monoculture and d3/d21 co-cultures, washed in PBS, resuspended in Opti-MEM (Fisher Scientific), and incubated at 37 ⁰C in 5% CO_2_ atmosphere for 24 h. After the incubation time, the CM were harvested, centrifuged, and the clear media were sterile filtered through 0.22 μm filters. ELISA was carried out on the CM samples using the Invitrogen mouse CCL2/MCP-1 uncoated and Invitrogen mouse VEGFA pre-coated plate kits (Thermo Fisher Scientific, Stone, UK), and the mouse CCL5/RANTES and IL-12 p70 DuoSet kits (R&D Systems, Abingdon, UK). The manufacturers’ protocols were followed. The concentrations of the cytokines and chemokines were normalised to the cell number of the monoculture.

**Fluorescence immunohistochemistry**

Tissue sections of snap-frozen, OCT-embedded s.c. B16F10 tumours were rehydrated, blocked with 1% horse serum (Fisher Scientific) and incubated with a rat anti-mouse CD11b-biotin antibody (clone M1/70, Fisher Scientific) followed by incubation with streptavidin-conjugated DyLight 594 (Fisher Scientific). The tissue sections were mounted using Fluoroshield mounting medium with DAPI (Sigma-Aldrich, Gillingham, UK), and visualised with an Olympus BX51 fluorescence microscope at 20x magnification (KeyMed Ltd., Southend-on-Sea, UK).

**Cytospin preparation**

From the sorted F4/80^+^CD11b^+^CD11c^-^ and the F4/80^+^CD11b^-^CD11c^+^ macrophages, 5x10^4^ cells were resuspended in 100 μl PBS containing 2% FBS and 0.05% Na-azide. The cell suspensions were loaded onto Thermo Scientific™ SuperFrost™ Plus adhesion slides (Fisher Scientific) and centrifuged at 600 RPM for 2 min using a Thermo Shandon Cytospin 3 cytocentrifuge (Marshall Scientific, Cambridge, USA). After centrifugation, the cells were dried on the slides and stained with 0.125% May-Grünwald and 0.04% Giemsa solutions (Sigma-Aldrich).

**Immunostaining for flow cytometry** (Details from main text)

To determine the expression of immune cell and proliferation markers in single cell suspensions of the lungs and in AMJ2-C11 mono- and co-cultures, the following anti-mouse monoclonal antibodies were applied: anti-CD45-FITC (clone 30-F11, Fisher Scientific), anti-F4/80-A647 (clone BM8, BioLegend, London, UK), anti-F4/80-PE (clone BM8, Fisher Scientific), anti-CD11b-FITC/PE-Cy7 (clone M1/70, Fisher Scientific), anti-CD11c-A647 and anti-CD11c-PE-Cy7 (clone N418, BioLegend and Fisher Scientific, resp.), anti-NK1.1-APC (clone PK136, Fisher Scientific), anti-Ly6G(Gr1)-PE (clone 1A8-Ly6g, Fisher Scientific), anti-Ly6C-APC (clone HK1.4, BioLegend), anti-CD3ε-PE-Cy7 (clone 145-2C11, Fisher Scientific), anti-CD206 (MR)-FITC (clone C068C2, BioLegend), anti-Ki-67-FITC/APC (clone 16A8, BioLegend), anti-CCR1-APC (clone #643854, R&D Systems), anti-CCR2-fluorescein/APC (clone #475301, R&D Systems) and anti-CCR5-PE/APC (clone HM-CCR5 (7A4), Fisher Scientific). Events were acquired using a BD FACSCalibur flow cytometer (BD, Wokingham, UK). The FlowJo v10.0.8 software (TreeStar, Ashland, USA) was used to analyse the data.

**Reference**

1 Van Hoecke L, Job ER, Saelens X, Roose K. Bronchoalveolar lavage of murine lungs to analyze inflammatory cell infiltration. *J Vis Exp* 2017; **123**: 55398.

**Table S1.** **Genes selected for a macrophage polarisation array (73 genes) and assays (6 chemokine receptor genes)**

| **Gene symbol – TaqMan accession number** | **Gene name** |
| --- | --- |
|  |  |
| **Arg1**-Mm01190441_g1 | arginase, liver |
| **Bax**-Mm00432050_m1 | BCL2-associated X protein (apoptosis regulator BAX) |
| **Bcl2**-Mm00477631_m1 | B cell leukemia/lymphoma 2 (apoptosis regulator Bcl-2) |
| **Bcl2l1**-Mm00437783_m1 | BCL2-like 1 (apoptosis regulator Bcl-X) |
| **C3**-Mm00437858_m1 | complement component 3 |
| **Ccl2**-Mm00441242_m1 | chemokine (C-C motif) ligand 2 (MCP-1) |
| **Ccl3**-Mm00441258_m1 | chemokine (C-C motif) ligand 3 (MIP-1-alpha) |
| **Ccl4**-Mm00443111_m1 | chemokine (C-C motif) ligand 4 (MIP-1-beta) |
| **Ccl5**-Mm01302428_m1 | chemokine (C-C motif) ligand 5 (RANTES) |
| **Ccl17**-Mm00516136_m1 | chemokine (C-C motif) ligand 17 |
| **Ccl20**-Mm00444228_m1 | chemokine (C-C motif) ligand 20 (MIP-3-alpha) |
| **Ccl22**-Mm00436439_m1 | chemokine (C-C motif) ligand 22 |
| **Ccr1**-Mm00438260_s1 | chemokine (C-C motif) receptor 1 |
| **Ccr2**-Mm99999051_gH | chemokine (C-C motif) receptor 2 |
| **Ccr3**-Mm01216172_m1 | chemokine (C-C motif) receptor 3 |
| **Ccr5**-Mm01216171_m1 | chemokine (C-C motif) receptor 5 |
| **Cxcr4**-Mm01292123_m1 | chemokine (C-X-C motif) receptor 4 |
| **Cx3cr1**-Mm02620111_s1 | chemokine (C-X3-C motif) receptor 1 |
| **Cxcl9**-Mm01345157_m1 | chemokine (C-X-C motif) ligand 9 (Mig) |
| **Cxcl10**-Mm00445235_m1 | chemokine (C-X-C motif) ligand 10 (IP-10) |
| **Cxcl11**-Mm00444662_m1 | chemokine (C-X-C motif) ligand 11 |
| **Cd14**-Mm00438094_g1 | Cd14 antigen (monocyte differentiation antigen) |
| **Cd38**-Mm00483146_m1 | Cd38 antigen (ADP-ribosyl cyclase 1) |
| **Cd40**-Mm00441895_m1 | CD40 antigen |
| **Cd68**-Mm00839636_g1 | CD68 antigen |
| **Cd86**-Mm00444543_m1 | CD86 antigen |
| **Cd163**-Mm00474091_m1 | Cd163 antigen (scavenger receptor cysteine-rich type 1 protein M130) |
| **Cd200r1**-Mm00491164_m1 | CD200 receptor 1 |
| **Chi3l3**-Mm00657889_mH | chitinase 3-like 3 (Ym1) |
| **Chi3l4**-Mm00840870_m1 | chitinase 3-like 4 (Ym2) |
| **Clec4a2**-Mm00488795_m1 | C-type lectin domain family 4, member a2 |
| **Clec7a**-Mm01183349 | C-type lectin domain family 7, member a |
| **Csf1**-Mm00432688_m1 | colony stimulating factor 1 (macrophage) |
| **Emr1**-Mm00802530_m1 | EGF-like module containing, mucin-like, hormone receptor-like sequence 1 (F4/80) |
| **F13a1**-Mm00472334_m1 | coagulation factor XIII, A1 subunit |
| **Fas**-Mm00433237_m1 | Fas (TNF receptor superfamily member 6) |
| **Fcgr1**-Mm00438874_m1 | Fc receptor, IgG, high affinity I |
| **Fn1**-Mm01256734_m1 | fibronectin 1 |
| **H2-Eb1**-Mm00439221_m1 | histocompatibility 2, class II antigen E beta |
| **Icam1**-Mm00516023_m1 | intercellular adhesion molecule 1 |
| **Il1a**-Mm00439620_m1 | interleukin 1 alpha |
| **Il1b**-Mm00434228_m1 | interleukin 1 beta |
| **Il6**-Mm00446190_m1 | interleukin 6 |
| **Il10**-Mm00439616_m1 | interleukin 10 |
| **Il12b**-Mm01288992_m1 | interleukin 12b (Il-12p40) |
| **Il15**-Mm00434210_m1 | interleukin 15 |
| **Il18**-Mm00434225_m1 | interleukin 18 |
| **Il1rn**-Mm00446185_m1 | interleukin 1 receptor antagonist |
| **Il4ra**-Mm01275139_m1 | interleukin 4 receptor, alpha |
| **Il27ra**-Mm00497259_m1 | interleukin 27 receptor, alpha |
| **Igf1**-Mm00439560_m1 | insulin-like growth factor 1 |
| **Ikbkb**-Mm00833995_m1 | inhibitor of kappaB kinase beta |
| **Irf1**-Mm00515191_m1 | interferon regulatory factor 1 |
| **Itgam**-Mm01271259_g1 | integrin alpha M (Cd11b) |
| **Itgax**-Mm00498698_m1 | integrin alpha X (Cd11c) |
| **Ly96**-Mm00444223_m1 | lymphocyte antigen 96 (MD-2) |
| **Lyve1**-Mm00475056_m1 | lymphatic vessel endothelial hyaluronan receptor 1 |
| **Mrc1**-Mm01329362_m1 | mannose receptor, C type 1 |
| **Nfkb1**-Mm00476361_m1 | nuclear factor of kappa light polypeptide gene enhancer in B cells 1 |
| **Nfkb2**-Mm00479807_m1 | nuclear factor of kappa light polypeptide gene enhancer in B cells 2 |
| **Nos2**-Mm00440485_m1 | nitric oxide synthase 2, inducible |
| **Ptgs2**-Mm00478374_m1 | prostaglandin-endoperoxide synthase 2 (Cox-2) |
| **Retnla**-Mm00445109_m1 | resistin like alpha (Fizz-1) |
| **Ski**-Mm00448744_m1 | ski sarcoma viral oncogene homolog |
| **Smad3**-Mm00489637_m1 | SMAD family member 3/ MAD homolog 3 |
| **Smad7**-Mm00484741_m1 | SMAD family member 7/ MAD homolog 7 |
| **Socs1**-Mm00782550_s1 | suppressor of cytokine signaling 1 |
| **Socs2**-Mm00850544_g1 | suppressor of cytokine signaling 2 |
| **Stab1**-Mm00460390_m1 | stabilin 1 |
| **Stat1**-Mm00439518_m1 | signal transducer and activator of transcription 1 |
| **Stat3**-Mm00456961_m1 | signal transducer and activator of transcription 3 |
| **Stat6**-Mm01160477_m1 | signal transducer and activator of transcription 6 |
| **Tek**-Mm00443243_m1 | endothelial-specific receptor tyrosine kinase (Tie2) |
| **Tgfb1**-Mm00441724_m1 | transforming growth factor, beta 1 |
| **Tlr2**-Mm00442346_m1 | toll-like receptor 2 |
| **Tlr4**-Mm00445274_m1 | toll-like receptor 4 |
| **Tnf**-Mm00443258_m1 | tumour necrosis factor |
| **Vcam1**-Mm00449197_m1 | vascular cell adhesion molecule 1 |
| **Vegfa**-Mm00437304_m1 | vascular endothelial growth factor A |

**Supplementary Figures**

**
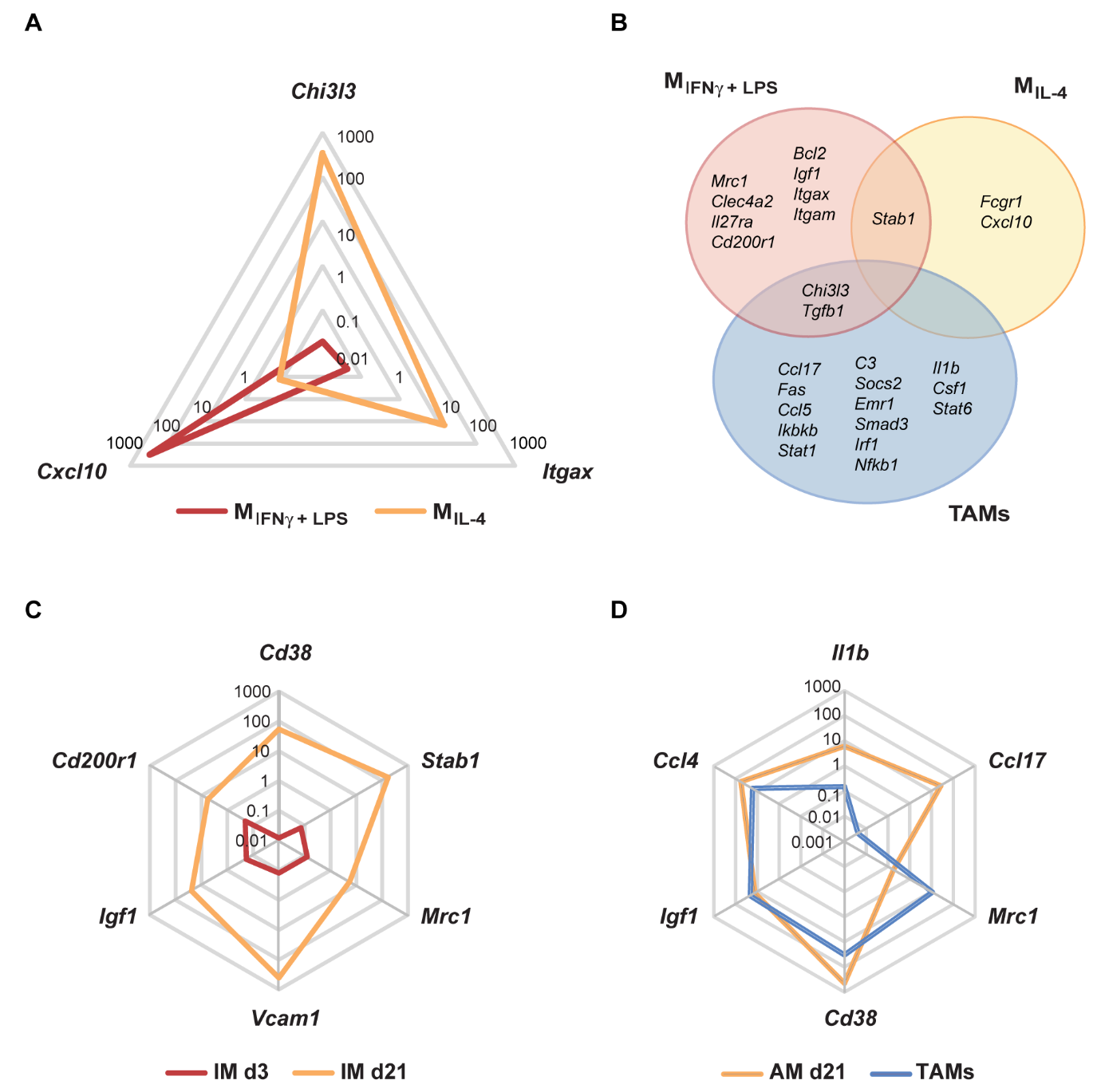
**

**Fig. S1: Comparison of gene expression of *in vitro* polarised macrophages, TAMs from s.c. melanoma and IM and AM from pulmonary metastasis**

Differentially regulated genes in M_IFN-γ+LPS_ and M_IL-4_, TAMs from s.c. melanoma, and IM and AM from lung metastasis were obtained via a customised PCR-array of macrophage polarity and immune response genes. **A** *Chi3l3*, *Itgax* and *Cxcl10* were found oppositely regulated in M_IFN-γ+LPS_ vs. M_IL4_. *Chi3l3* and *Itgax* were upregulated in M_IL-4_ whereas downregulated in M_IFN-γ+LPS_. On the other hand, *Cxcl10* was upregulated in M_IFN-γ+LPS_ whereas downregulated in M_IL-4_. **B** Venn diagram showing the overlap of downregulated genes between M_IFN-γ+LPS_, M_IL-4_ and TAMs. **C** Six genes, including *Mrc1*, *Cd38* and *Igf1* (TAMs markers), were found oppositely regulated in IM at the early (d3) vs. late stage (d21) of metastatic growth. All six genes were downregulated at d3 whereas upregulated at d21. **D** Gene regulation showing similarities and differences alike in AM at d21 vs. TAMs. *Ccl4*, *Igf1* and *Cd38* were upregulated both in AM and TAMs. *Il1b* and *Ccl17* were downregulated in TAMs whereas upregulated in AM. *Mrc1* was upregulated in TAMs but downregulated in AM.

**
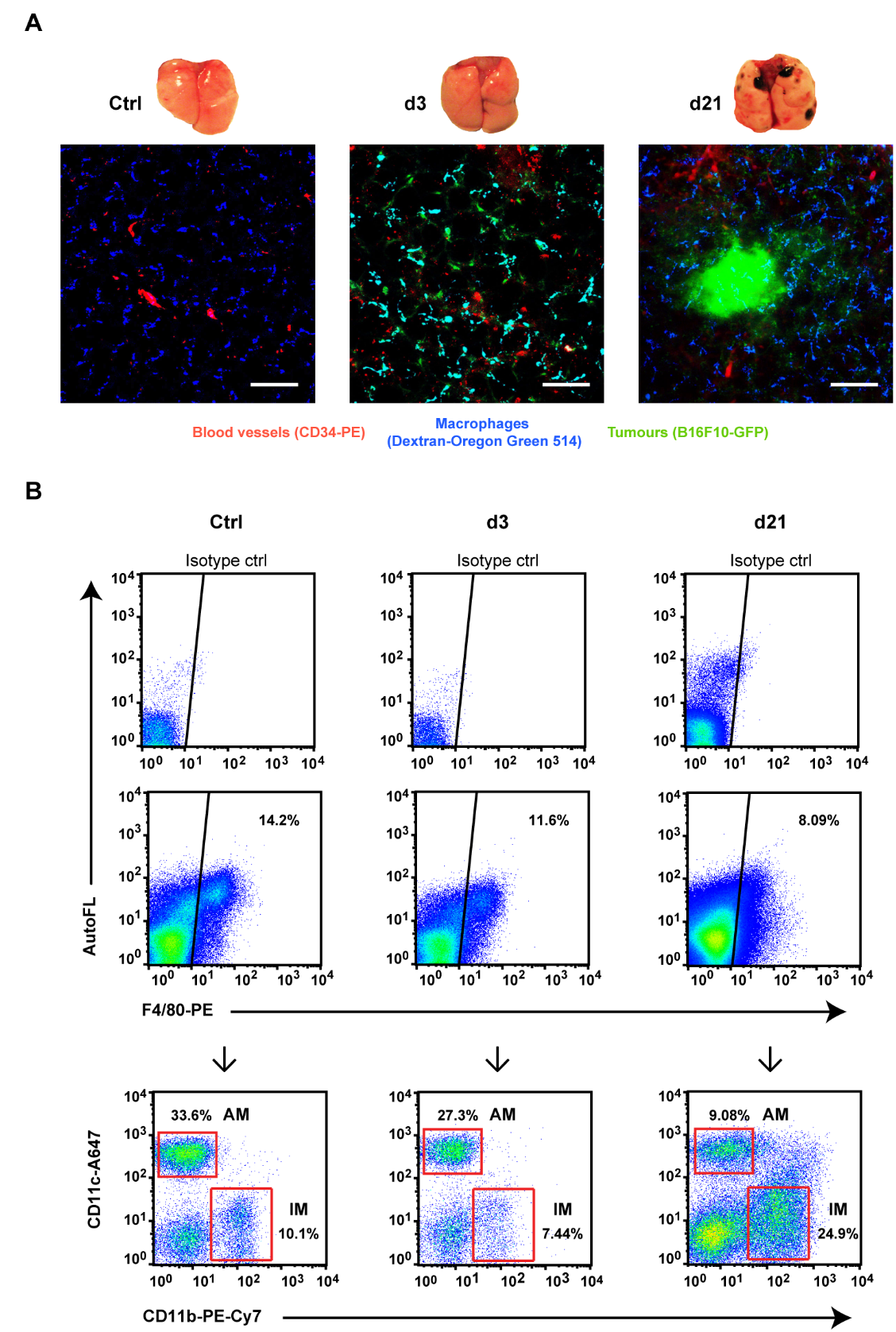
**

**Fig. S2: Gating strategy for fluorescence activated cell sorting of IM and AM at the early or late stage of pulmonary metastasis**

**A** B16F10-GFP cells were injected into the tail vein of C57BL/6 mice, and the lungs were harvested at d3 or d21 after tumour challenge. Twenty-four hours before sacrifice, mice were i.v. injected with Dextran-Oregon Green 514 to visualise macrophages, and 1 hour before sacrifice the mice were also injected with a PE-conjugated anti-CD34 antibody to detect endothelial cells. The harvested lungs were imaged using confocal microscopy. Representative photos of the lungs at harvest and the corresponding confocal images are shown where macrophages labelled with Oregon Green 514 are blue, GFP-expressing tumour cells are green and CD34-PE-labelled endothelial cells are red. Size bars indicate 50 μm. **B** Representative dot plots are shown of F4/80-gated CD11b^+^CD11c^-^ (IM) and CD11b^-^CD11c^+^ (AM) cells sorted from control (unchallenged) lungs or metastasis-bearing lungs at d3 or d21.

**
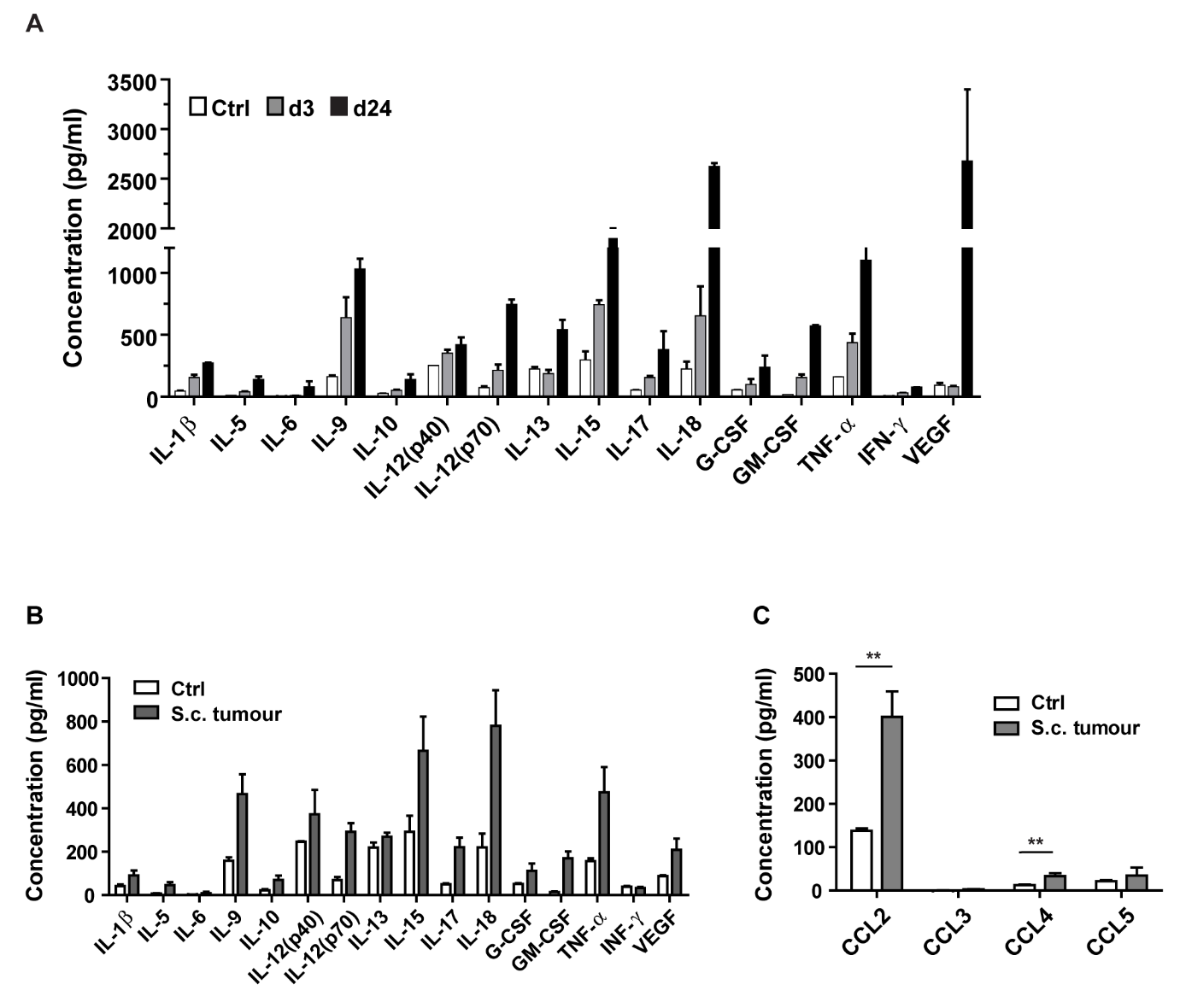
**

**Fig. S3: Cytokine and chemokine secretion profiles from the sera of mice with pulmonary metastasis or s.c. melanoma**

**A-C** Blood was taken via cardiac puncture from unchallenged (control) mice (n=3), from lung metastasis-bearing mice at d3 or d24 (n=5 per group) (**A**)*,* and from s.c. B16F10 melanoma-bearing mice (n=5) (**B, C**). The sera were subjected to Luminex assay to determine cytokine and chemokine levels. Bars represent the mean concentrations + SD. To assess the differences between the means, unpaired t-tests were performed, ***p<0.01* (**C**).


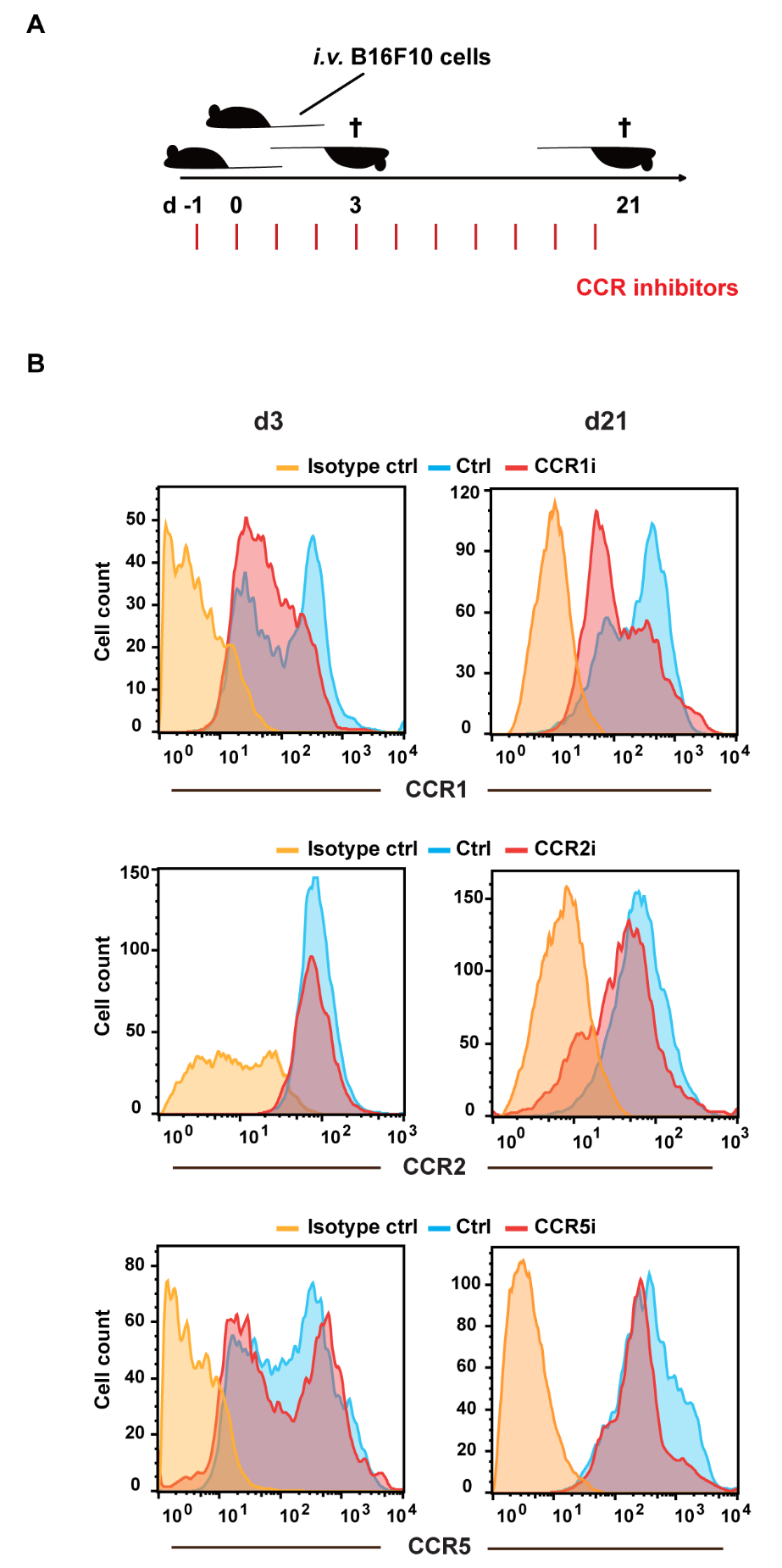


**Fig. S4: CCR1, CCR2 and CCR5 expression of lung macrophages in response to chemokine receptor inhibition**

**A** C57BL/6 mice were injected with 2x10^5^ B16F10 cells into the tail vein. Daily doses of chemokine receptor antagonists targeting CCR1 (J-113863/CCR1i), CCR2 (RS-504393/CCR2i) or CCR5 (DAPTA/CCR5i) were administered from the day before tumour cell injection. Control mice were left untreated. The antagonist treatment continued until lung harvest at d3 or d21. **B** CCR1, CCR2 and CCR5 cell surface expression was determined in F4/80^+^ lung macrophages in response to the respective CCRi at d3 and d21 by flow cytometry. Representative histograms of the isotype controls, the untreated lungs (control) and the CCRi-treated lungs are shown at d3 and d21.


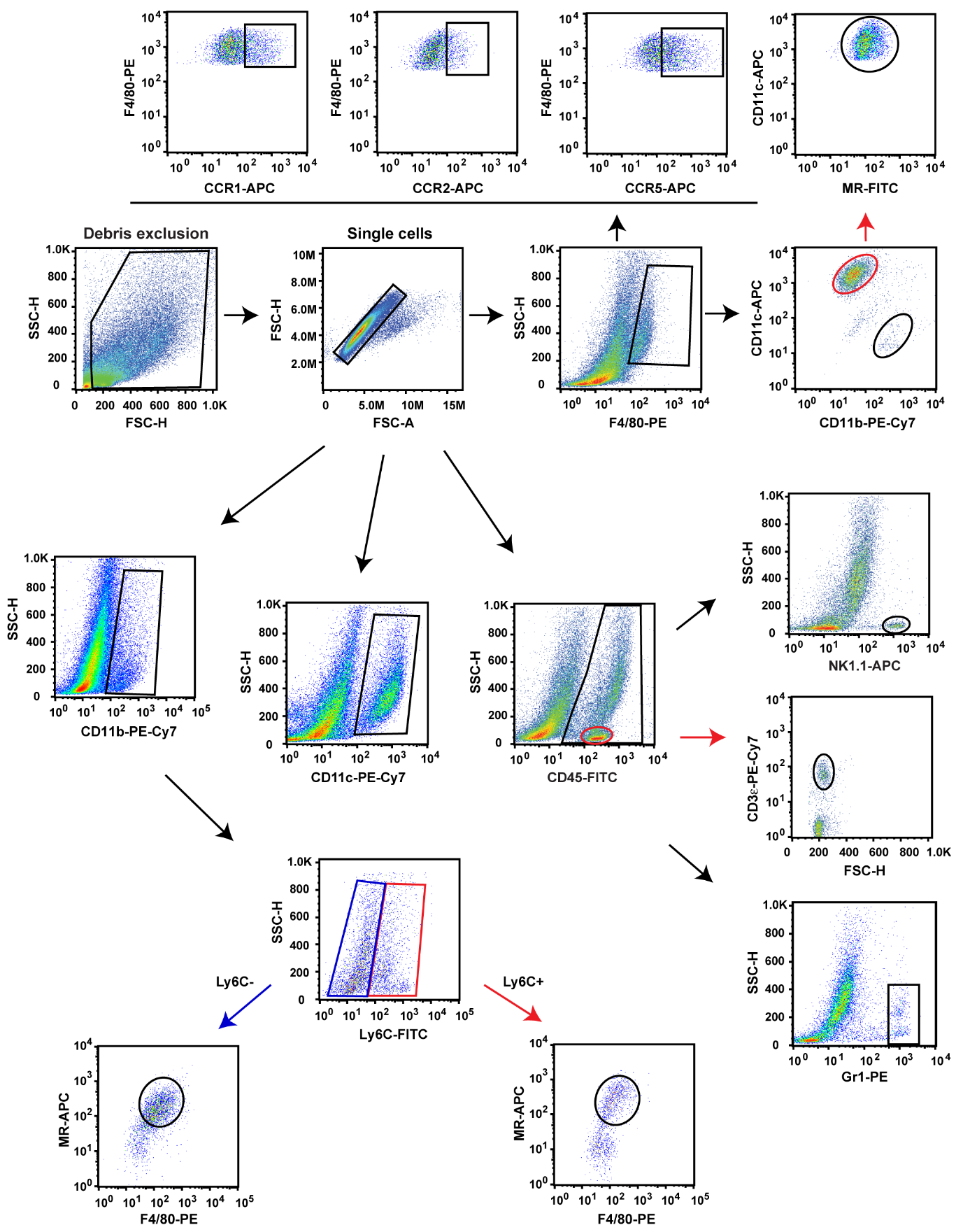


**Fig. S5: Gating strategy used in flow cytometry analysis of immune cells in the lungs**

In single cell suspensions of the lungs, FSC vs. SSC gating was used for debris exclusion, and FSC-A vs. FSC-H gating for aggregate exclusion (single cells). Within the CD45 gate (leukocytes), we used F4/80 gating to select all macrophages, and within the F4/80 gate CD11b and CD11c served as IM and AM markers, respectively, AM also being MR^+^. Chemokine receptor positivity of macrophages was determined on the F4/80^+^ gate. NK1.1 was used as a NK cell marker, Gr1 (Ly6G) as a granulocyte (neutrophil) marker and CD3ε as a lymphocyte marker. At d21, the pro-inflammatory macrophages were identified as the F4/80^+^MR^+^ subset on the CD11b^+^ and Ly6C^+^ gates, whereas the anti-inflammatory/pro-tumour macrophages as the F4/80^+^MR^+^ subset on the CD11b^+^ and Ly6C^-^ gates.


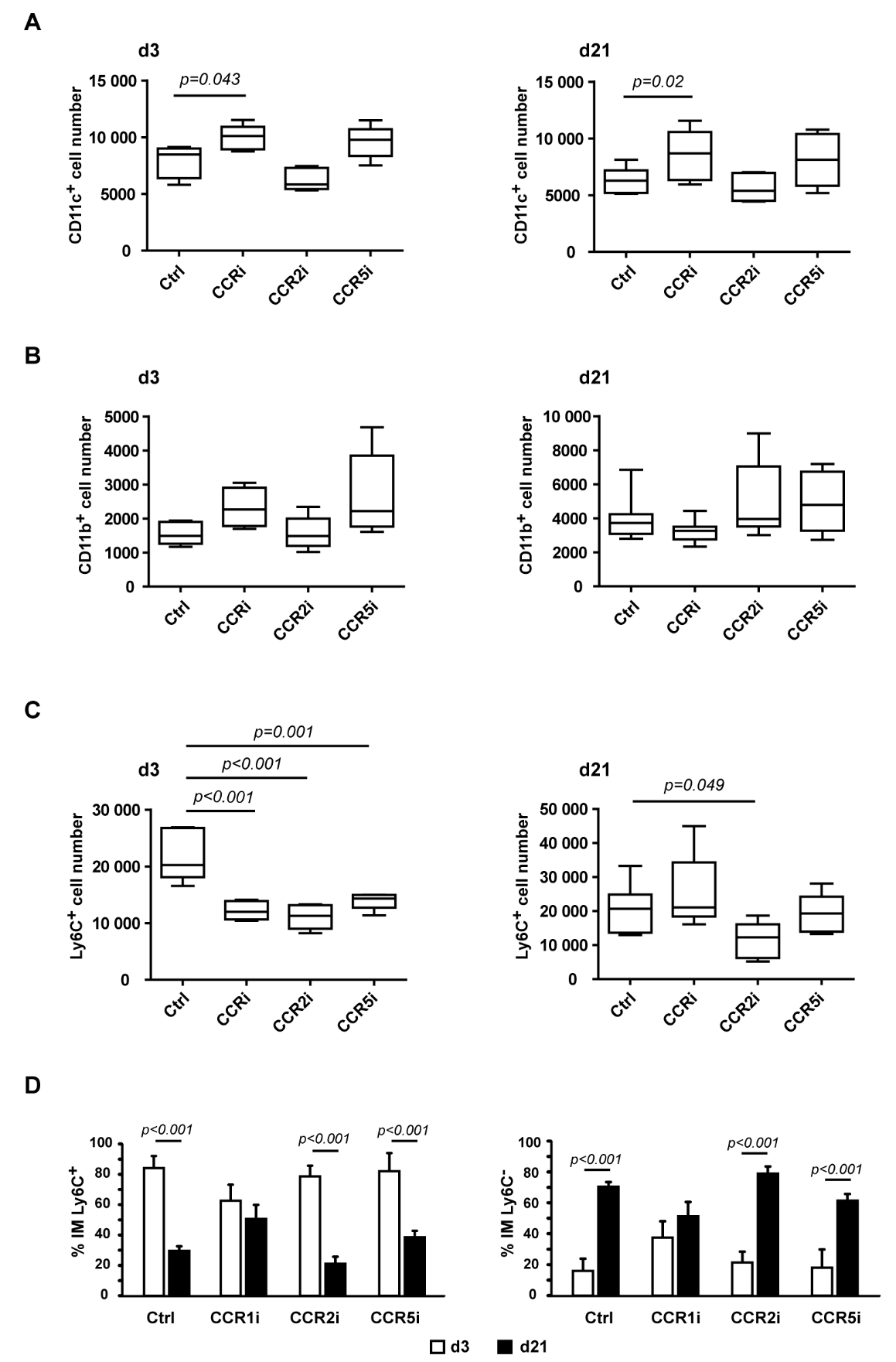


**Fig. S6: The total CD11c^+^, CD11b^+^ and Ly6C^+^ cell populations of IM in the lungs in response to chemokine receptor inhibition during metastasis progression**

Single cell suspensions of lungs from control (untreated) and CCRi-treated mice at d3 or d21 were subjected to flow cytometry analysis to determine the frequency of CD11c, CD11b and Ly6C-expressing cells in 10^5^ total viable events, n=5 at both d3 and d21. **A-C** Boxplots indicate the total numbers of CD11c^+^ (**A**), CD11b^+^ (**B**) and Ly6C^+^ (**C**) cells ± SD. To assess the differences between the means, one-way ANOVA and Dunnett’s multiple comparison tests were conducted. **D** In the IM population, the Ly6C^+^ and Ly6C^-^ subsets were quantified and expressed as percentages (%) of the total IM numbers. The bars show the mean % of Ly6C^+^ or Ly6C^-^ IM + SD, n=5. The differences between the means were assessed by unpaired t-tests.


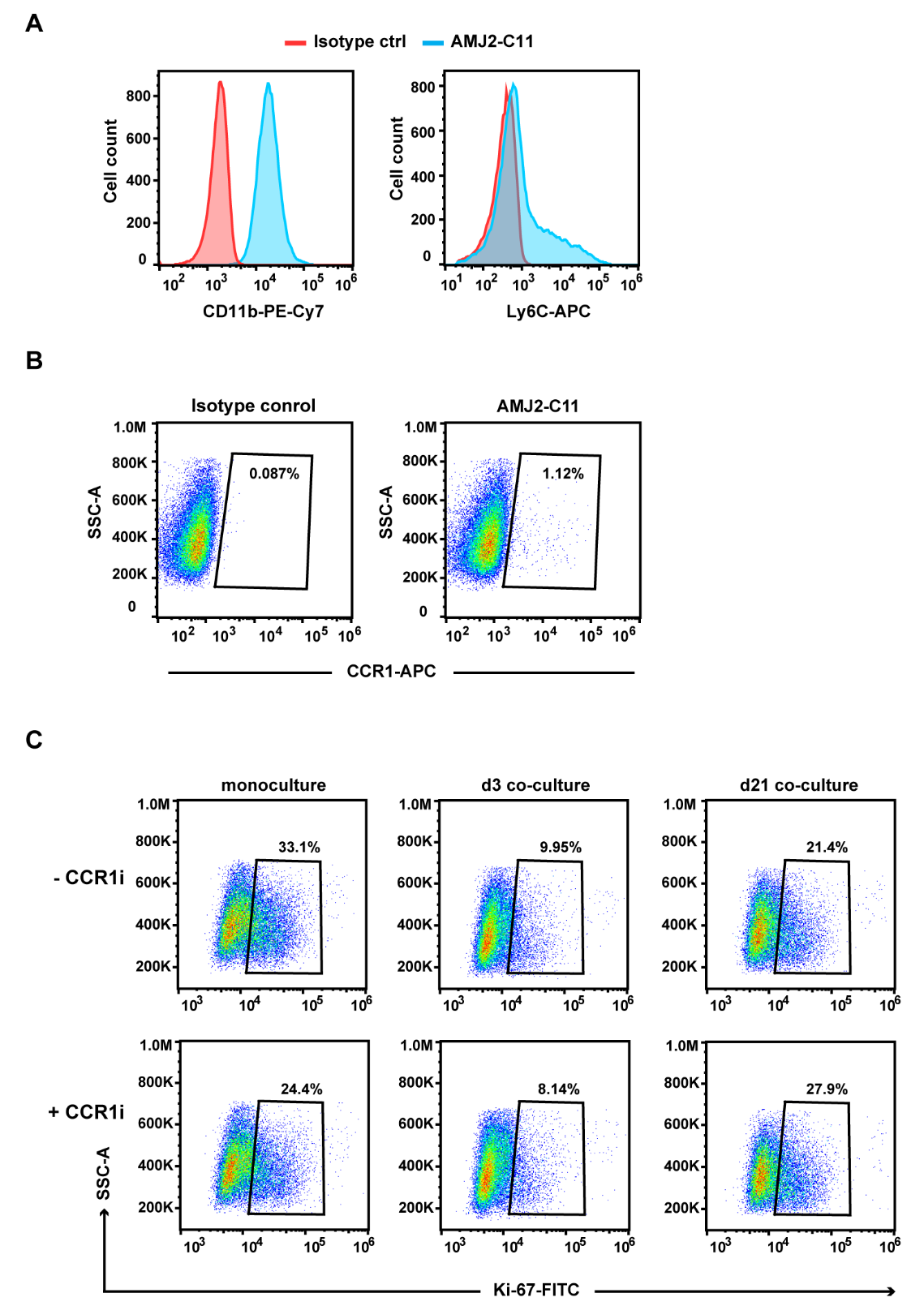


**Fig. S7: Proliferation of AMJ2-C11 macrophages in monoculture or co-culture in response to CCR1i**

Mono- and co-cultured AMJ2-C11 mouse macrophages were subjected to flow cytometry analysis to determine their CD11b, Ly6C, CCR1 and Ki-67 expression. **A** Representative histograms demonstrating that all AMJ2-C11 cells were CD11b^+^, and >30% of the cells also expressed Ly6C. **B** Representative dot plots showing that AMJ2-C11 cells have a low level of CCR1 expression. **C** AMJ2-C11 monoculture or co-culture with B16F10 cells for up to 21 days were treated with CCR1i for 48 h, or left untreated. Flow cytometry analysis conducted to determine Ki-67 expression indicates that CCR1i does not have a significant impact on the proliferation of co-cultured AMJ2-C11 cells.


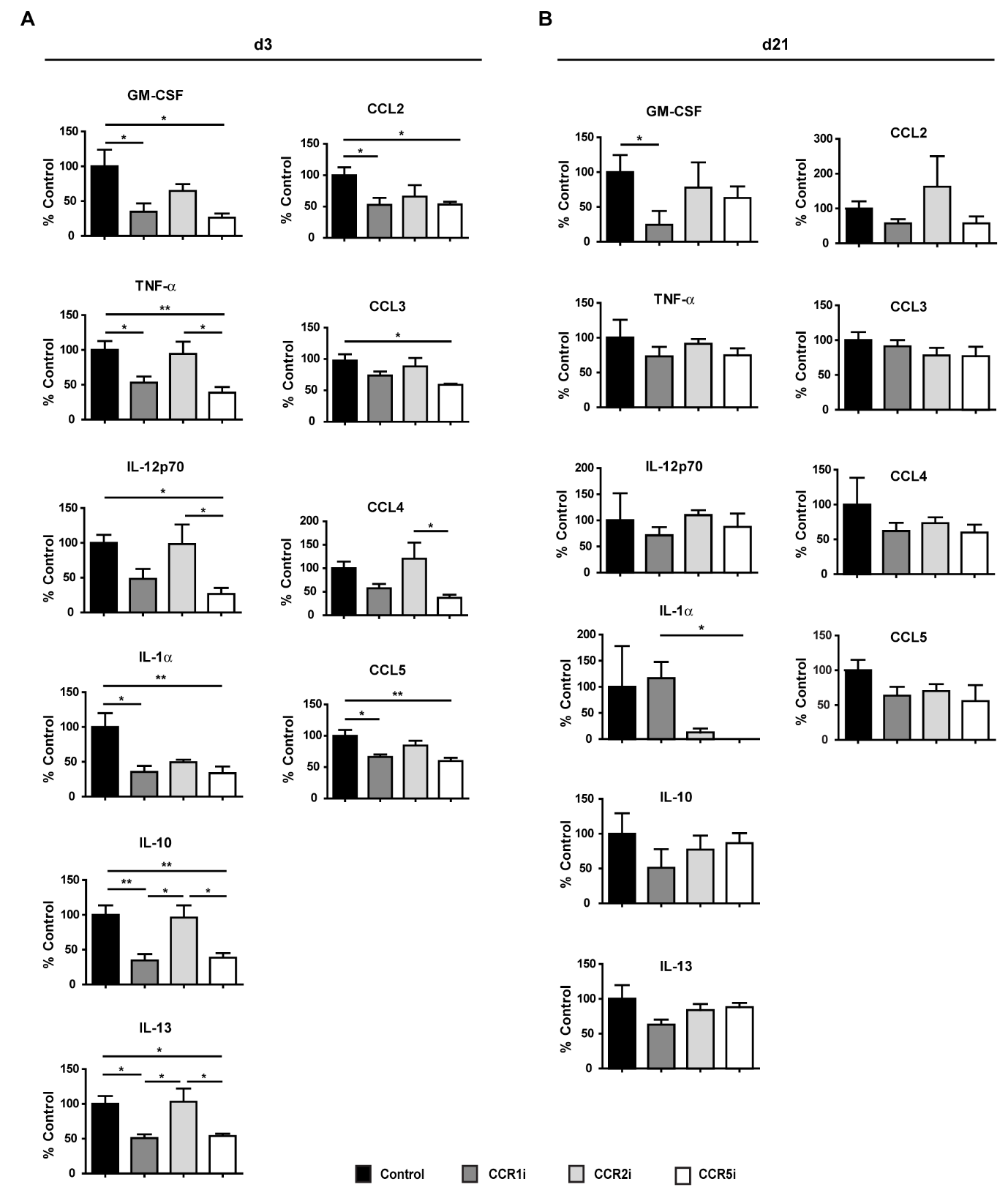


**Fig. S8: Serum cytokine and chemokine levels in response to chemokine receptor antagonists during metastasis progression**

**A, B** Blood was taken via cardiac puncture from mice at d3 (**A**) or d21 (**B**) after i.v. injection of B16F10 cells, treated or not (control) with CCR1i, CCR2i or CCR5i. Serum concentrations of cytokines and chemokines were determined by Luminex assay. The results are shown as concentrations relative to the control (% Control). The bars represent the mean relative concentrations + SD, n=4-5 per group. To determine the differences between the means, one-way ANOVA and Tukey’s pairwise comparison (for normally distributed data) or Kruskal-Wallis and Bonferroni’s post-hoc tests (for not normally distributed data) were carried out, **p<0.05*, ***p<0.01*.
